# Release of condensin from mitotic chromosomes requires the Ran-GTP gradient in the reorganized nucleus

**DOI:** 10.1242/bio.027193

**Published:** 2017-09-27

**Authors:** Keita Aoki, Hironori Niki

**Affiliations:** 1 Microbial Genetics Laboratory, Genetic Strains Research Center, National Institute of Genetics, 1111 Yata, Mishima, Shizuoka 411-8540, Japan; 2 Department of Genetics, SOKENDAI, National Institute of Genetics, 1111 Yata, Mishima, Shizuoka 411-8540, Japan

**Keywords:** Pim1/RCC1, Condensin, Chromosome decondensation

## Abstract

After mitosis, nuclear reorganization occurs together with decondensation of mitotic chromosomes and reformation of the nuclear envelope, thereby restoring the Ran-GTP gradient between the nucleus and cytoplasm. The Ran-GTP gradient is dependent on Pim1/RCC1. Interestingly, a defect in Pim1/RCC1 in *Schizosaccharomyces pombe* causes postmitotic condensation of chromatin, namely hypercondensation, suggesting a relationship between the Ran-GTP gradient and chromosome decondensation. However, how Ran-GTP interacts with chromosome decondensation is unresolved. To examine this interaction, we used *Schizosaccharomyces japonicus*, which is known to undergo partial breakdown of the nuclear membrane during mitosis. We found that Pim1/RCC1 was localized on nuclear pores, but this localization failed in a temperature-sensitive mutant of Pim1/RCC1. The mutant cells exhibited hypercondensed chromatin after mitosis due to prolonged association of condensin on the chromosomes. Conceivably, a condensin-dephosphorylation defect might cause hypercondensed chromatin, since chromosomal localization of condensin is dependent on phosphorylation by cyclin-dependent kinase (CDK). Indeed, CDK-phospho-mimic mutation of condensin alone caused untimely condensin localization, resulting in hypercondensed chromatin. Together, these results suggest that dephosphorylation of CDK sites of condensin might require the Ran-GTP gradient produced by nuclear pore-localized Pim1/RCC1.

## Abstract (precis)

**Summary:** A mutant of Pim1/RCC1 caused hypercondensed chromatin after mitosis due to prolonged association of condensin on chromosomes, suggesting that dephosphorylation of CDK sites of condensin might require Ran-GTP after mitosis.

## INTRODUCTION

In the open mitosis of higher eukaryotic cells, nuclear envelope breakdown and fragmentation along mitotic chromosomes occur before chromosome segregation (Guttinger et al., 2009; Kutay and Hetzer, 2008; Sazer, 2005). During G1 phase, the fragmented nuclear envelope is fused, leading to reformation of the nuclear envelope and the gradient of Ran-GTP between the nucleus and cytoplasm. Formation of the Ran-GTP gradient is largely dependent on RCC1, a Ran guanine nucleotide exchange factor. RCC1 (Kai et al., 1986; Bischoff and Ponstingl, 1991) is localized on chromatin (Ohtsubo et al., 1989). RanGAP, a RanGTPase-activating protein (Bischoff et al., 1995), is localized in the cytoplasm (Feng et al., 1999). Therefore, a gradient of Ran-GTP is formed between the nucleus and cytoplasm (Kalab et al., 2002). The gradient is important for Ran-mediated biological functions such as nucleocytoplasmic transport, spindle formation and fusion of the nuclear membrane (Kahana and Cleveland, 1999; Clarke and Zhang, 2001; Gruss et al., 2001; Dasso, 2002; Hetzer et al., 2002). The catalytic activity of RCC1 is derived from the seven-bladed propeller structure of the RCC1 repeats (Ohtsubo et al., 1989; Renault et al., 1998). Formation of the nuclear envelope and the Ran-GTP gradient are accompanied by the decondensation of mitotic chromosomes during G1 phase, resulting in reorganization of the daughter nuclei (Guttinger et al., 2009; Kutay and Hetzer, 2008; Sazer, 2005). However, how the nuclear envelope and Ran-GTP affect the decondensation of chromosomes is not fully understood.

Several studies have reported the untimely condensation of chromatin due to dysfunction of RCC1 homologs. In hamster, a temperature-sensitive BN2 cell, which is defective in RCC1 function, has been shown to exhibit prematurely condensed chromosomes and fragmented nuclei (Nishimoto et al., 1978). In *Schizosaccharomyces* *pombe*, a mutant of Pim1 that is a homolog to RCC1 exhibited the condensed chromatin (Matsumoto and Beach, 1991; Sazer and Nurse, 1994; Hirose et al., 2006). A study using *S. pombe* showed that when Pim1 is dysfunctional, the cells undergo arrest, exhibiting a medial septum and dual nuclei with condensed chromatin. The binucleated cells accumulate with a 1C DNA content per nucleus, indicating that the cells do not undergo the subsequent S phase in the *pim1* mutant. Therefore, the activity of Pim1 was shown to affect the decondensation of mitotic chromosomes, and the decondensation preceded progression of the nuclear cycle during the S phase (Sazer and Nurse, 1994). These findings were supported by a recent study using a cell-free assay, in which chromosome decondensation was shown to require GTP hydrolysis (Magalska et al., 2014).

In addition, the condensed chromatin does not always occur in both of the nuclei in a binucleated cell in *S. pombe* (Hirose et al., 2006; Gonzalez et al., 2009). The mis-segregation of chromosomal DNA in *pim1* mutant was caused by a defect of mitotic spindle formation (Hirose et al., 2006), and the condensed chromatin was consistently associated with the newer spindle pole body in *pim1-d1* mutant in *S. pombe* (Gonzalez et al., 2009). Moreover, the condensed chromatin has also been observed in a deletion mutant of the nuclear pore complex in *S. pombe* (Bai et al., 2004). Despite numerous investigations, however, how the chromosome fails in decondensation in the *pim1* mutant during the G1 phase has not been adequately clarified.

The mitotic condensation of chromosomes is largely dependent on the activity of condensin (Hirano, 2004, 2016; Yanagida, 2005), which is localized on chromosomes in a manner dependent on the phosphorylation of a structural maintenance of chromosomes (SMC) subunit of condensin by cyclin-dependent kinase (CDK) during mitosis (Sutani et al., 1999; Nakazawa et al., 2008). Conversely, when the chromosomes are decondensed, the condensin is dissociated from mitotic chromosomes in a manner dependent on dephosphorylation of the SMC subunit of condensin (Sutani et al., 1999). However, how the release of condensin is involved in the Ran-GTP is not fully understood.

We considered that *Schizosaccharomyces japonicus*, a fission yeast that undergoes a semi-open mitosis, would be an advantageous model for investigating the involvement of Ran-GTP in condensin release. The semi-open mitosis would enable us to observe the ruptured and reformed nuclear envelope during late mitosis (Robinow and Hyams, 1989; Aoki et al., 2011; Yam et al., 2011), which is accompanied by a change of the Ran-GTP gradient between the nucleus and cytoplasm, and thus to investigate how the gradient of Ran-GTP affects the decondensation of mitotic chromosomes. Indeed, we anticipate that studies using semi-open mitosis will provide new insights in the field of nuclear division that were unavailable in the previous studies using closed mitosis (Walters et al., 2012; Boettcher and Barral, 2013).

## RESULTS

### Hypercondensed chromatin was produced in a *pim1* mutant

**Fig. 1.**
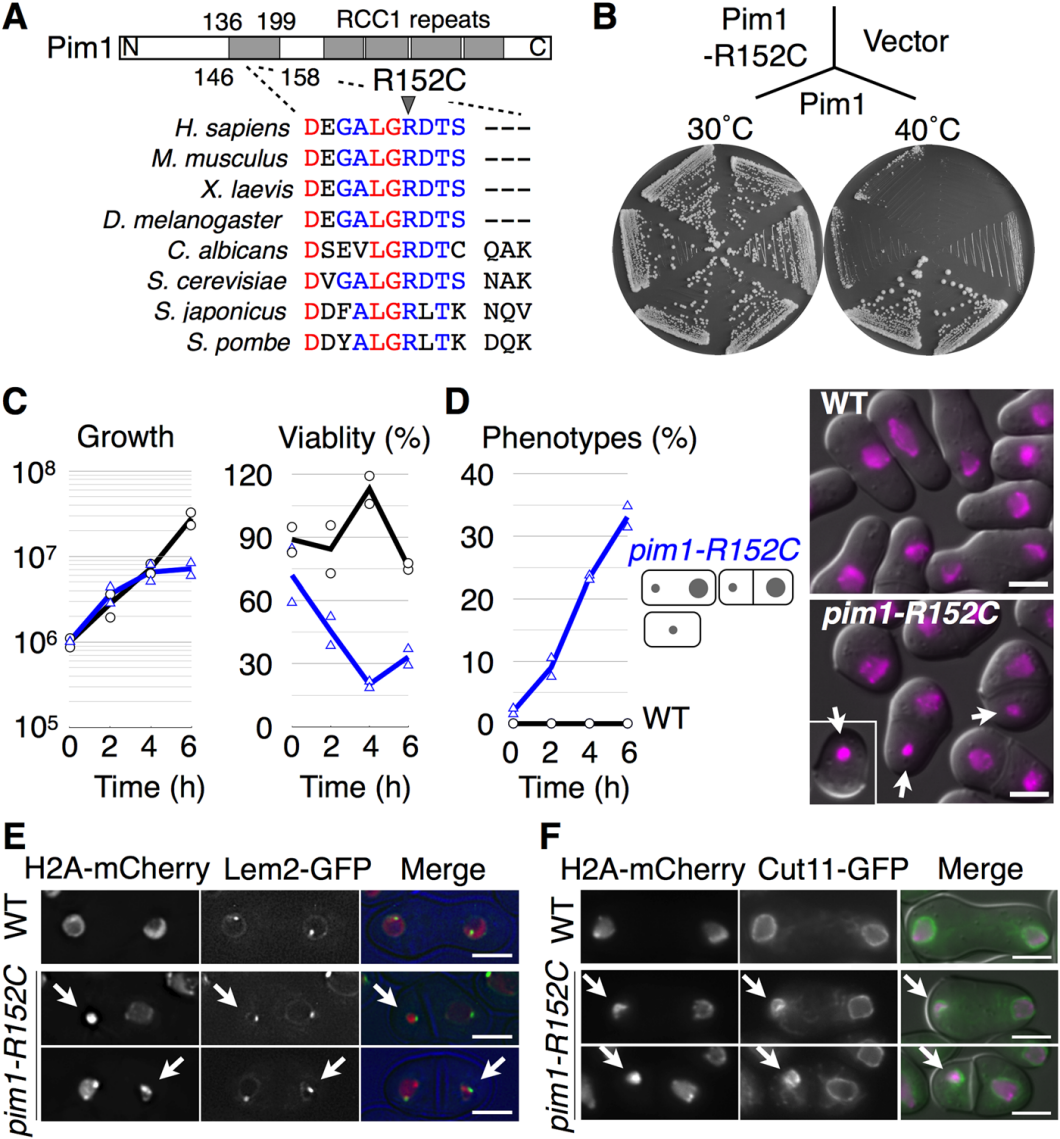
**Decondensation of chromatin is inhibited in a temperature-sensitive mutant of *pim1-R152C*.** (A) Homologous sequences of each RCC1 corresponding to the region from aspartic acid 146 to valine 158 in *S. japonicus* Pim1 are presented. (B) The growth defect of NIG8002 was restored in transformants with pSJU11-*pim1*, but not pSJU11-*pim1^R152C^* or pSJU11 (Vector), in EMM2 medium. (C) Growth rates and viabilities were observed in the WT (circles) and *pim1-R152C* (triangles). NIG2028 and NIG8001 were cultivated in YE (+Ade, Ura) medium to log phase at 30°C, then shifted to 37°C for 6 h. (D) NIG8003 was cultivated as in C to observe nuclear phenotypes at 0 h (*n*=228), 2 h (*n*=167), 4 h (*n*=229) and 6 h (*n*=199). NIG8004 was cultivated as in C to observe nuclear phenotypes at 0 h (*n*=151), 2 h (*n*=239), 4 h (*n*=244) and 6 h (*n*=253). Hypercondensed chromatin was accumulated in binucleated cells or one-nucleus cells of *pim1-R152C* (triangles), but not in the WT (circles). In both C and D, each symbol shows results from two independent experiments, and the line graphs show the mean of these experiments. Nuclear phenotypes painted by H2A-mCherry are described. Arrows indicate hypercondensed chromatin. (E) Localization of H2A-mCherry (red) and Lem2-GFP (green) was observed in the WT (*n*=238) and *pim1-R152C* (*n*=271). NIG8006 and NIG8007 were cultivated as in C for 2 h then observed using a DeltaVision microscope. 91.4% of the hypercondensed chromatin (*n*=35) was surrounded by the Lem2-GFP. Arrows indicate hypercondensed chromatin with Lem2-GFP. The DIC images (blue) were also merged. (F) Localization of H2A-mCherry (magenta) and Cut11-GFP (green) was observed in the WT (*n*=250) and *pim1-R152C* (*n*=363). NIG8896 and NIG8005 were cultivated as in E then observed. 87.8% of the hypercondensed chromatin (*n*=49) was surrounded by the Cut11-GFP. Arrows indicate hypercondensed chromatin with Cut11-GFP. Two independent experiments were performed in each analysis. Scale bars: 5 µm.

In order to understand how cells decondense their chromatin, we isolated a mutant having ‘hypercondensed chromatin’, which we defined as a chromosome condensed in the post-anaphase state, as described previously (Sazer and Nurse, 1994). A mutant of ts581, which exhibited hypercondensed chromatin by this definition, was isolated from a temperature-sensitive mutant library of *S. japonicus* (Aoki et al., 2013). To uncover the causative gene of ts581, whole-genome sequencing and genetic analyses were performed, revealing that *pim1^+^* (SJAG_04464.5), a homolog of RCC1, was a causative gene for the temperature sensitivity of ts581. The mutated Pim1 had an amino acid substitution of R152C (Fig. 1A) and was designated *pim1-R152C*. The 152nd arginine of Pim1 was located in an RCC1 repeat and was broadly conserved from yeast to humans (Fig. 1A). The colony formation ability of *pim1-R152C* was suppressed by exogenous expression of Pim1, but not by exogenous expression of Pim1^R152C^ or a control vector, on agar plates at 40°C, indicating that the R152C mutation of Pim1 was recessive (Fig. 1B).

To investigate the mutant phenotypes of *pim1-R152C* at restrictive temperature in live cells, the growth rate and cell viability were observed at 37°C for 6 h (Fig. 1C,D). The viability of *pim1-R152C* was severely decreased 4 h after shifting the cells from 30°C to 37°C, accompanied by delayed cell growth (Fig. 1C). To observe the chromosomal morphology of *pim1-R152C*, localization of H2A-mCherry was observed at 37°C for 6 h (Fig. 1D). H2A-mCherry is a fluorescently labeled version of the histone protein H2A used to visualize the chromosomes. The hypercondensed chromatin (arrows) was totally accumulated in 33.1% of cells in *pim1-R152C* at 6 h after shifting the cells from 30°C to 37°C, as shown by the blue line in Fig. 1D.

### Hypercondensed chromatin was surrounded by a nuclear envelope

To examine whether or not the hypercondensed chromatin was surrounded by a nuclear envelope, we observed the localization of GFP-AHDL, which represents the lipid membrane (Yam et al., 2011), GFP-fused Lem2, which represents the inner nuclear membrane or spindle pole body (Holmer and Worman, 2001; Hiraoka et al., 2011), and GFP-fused Cut11, which represents the nuclear pore complex (West et al., 1998) in wild type (WT) and *pim1-R152C* at 37°C for 2 h. We found that the hypercondensed chromatin was surrounded by signals of the GFP-AHDL, Lem2-GFP or Cut11-GFP, as shown by the arrows in Fig. 1E and F, and Fig. S1A, respectively. In addition, dot-like signals of the Lem2-GFP, which represent the localization of SPB, were also observed around the hypercondensed chromatin (Fig. 1E). However, the nuclear envelope around the hypercondensed chromatin would be ruptured or nonfunctional because the Ran-GTP gradient is not retained (Fig. 3B,E). These results indicate that the hypercondensed chromatin was surrounded by a nonfunctional nuclear envelope with both nuclear pore complexes and spindle pole bodies.

### Pim1 was localized on the nuclear pore complex in a manner independent of chromosomes

**Fig. 2.**
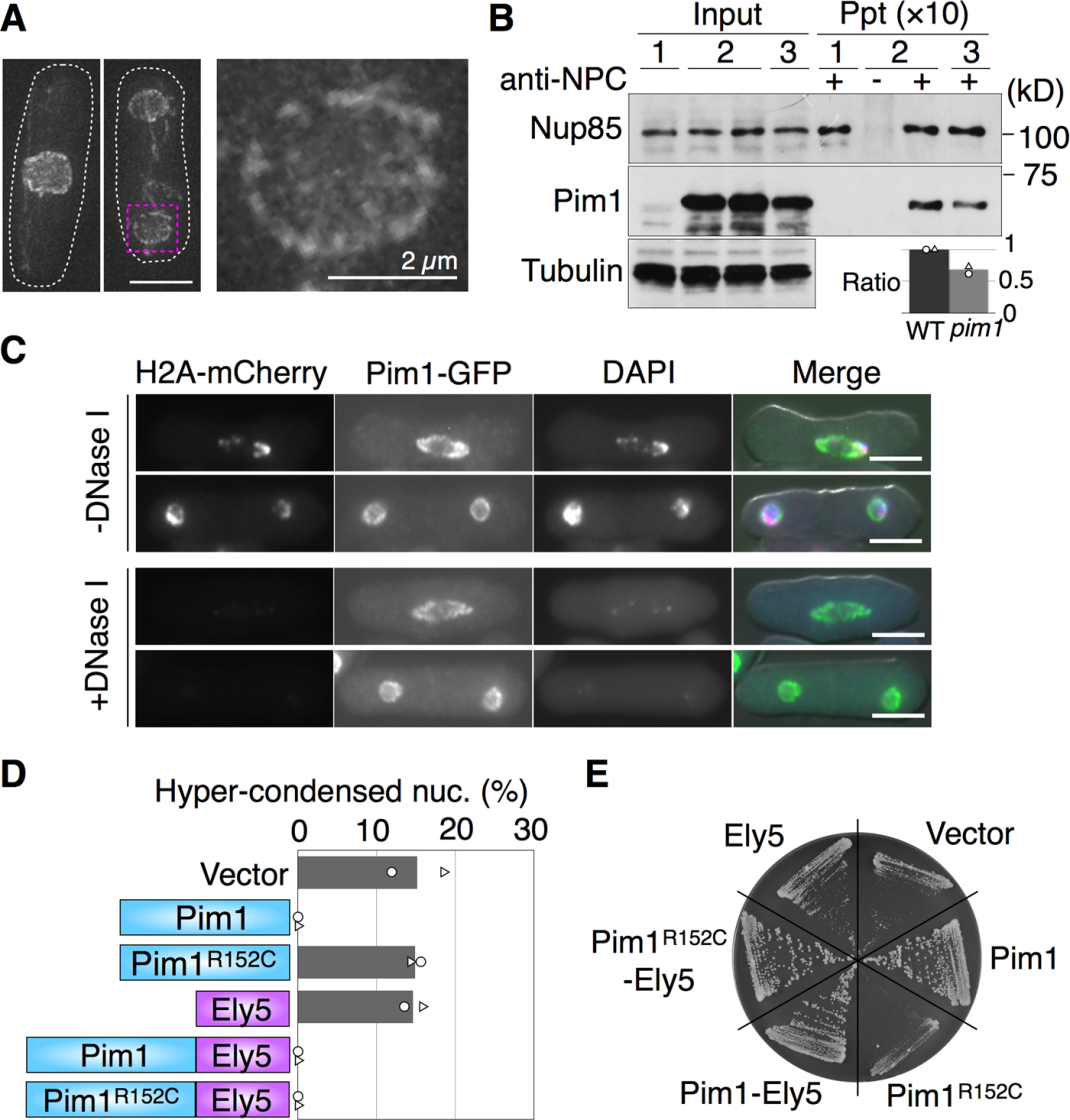
**Pim1 is localized on the nuclear pore complex independent of the chromosome.** (A) NIG8031 was cultivated to log phase in YE (+Ade, Ura) medium at 30°C and observed using a DeltaVision microscope (*n*=33). 50 images of Pim1-GFP were taken at 0.1 µm intervals along the z-axis and deconvolved. White dashed lines indicate the cellular shape. A nucleus surrounded by the magenta dashed line was enlarged. (B) Immunoprecipitation with a nuclear pore complex antibody (Mab414) was performed using NIG8010 (lane 1), NIG8011 (lane 2) and NIG8012 (lane 3). The ratio represents the comparison of immunoprecipitated Pim1-3Flag signals in the WT and *pim1-R152C*, which were normalized by immunoprecipitated Nup85-GFP signals and tubulin signals. (C) An *in situ* chromatin-binding assay using NIG8013 was performed with (*n*=180) or without (*n*=275) DNase. Green, Pim1-GFP; magenta, H2A-mCherry; blue, DAPI. DIC images were merged. (D) Transformants of NIG8039 with pSJU11 (vector), pSJU11-*pim1*, pSJU11-*pim1^R152C^*, pSJU11-*ely5*, pSJU11-*pim1-ely5*-*GFP* or pSJU11-*pim1^R152C^-ely5-GFP* were cultivated to log phase in YE (+Ade, Ura) medium at 30°C, then shifted to 37°C for 2 h, to observe nuclear phenotypes. The numbers of the hypercondensed chromatin stained by H2A-mCherry were counted under an AxioVision microscope. (E) Colony formation ability of these transformants was examined on YE (+Ade, Ura) plates at 37°C. In both B and D, each symbol shows results from two independent experiments, and the bar graphs show the mean of these experiments. Two independent experiments were performed in each analysis. Scale bars: 5 µm.

**Fig. 3.**
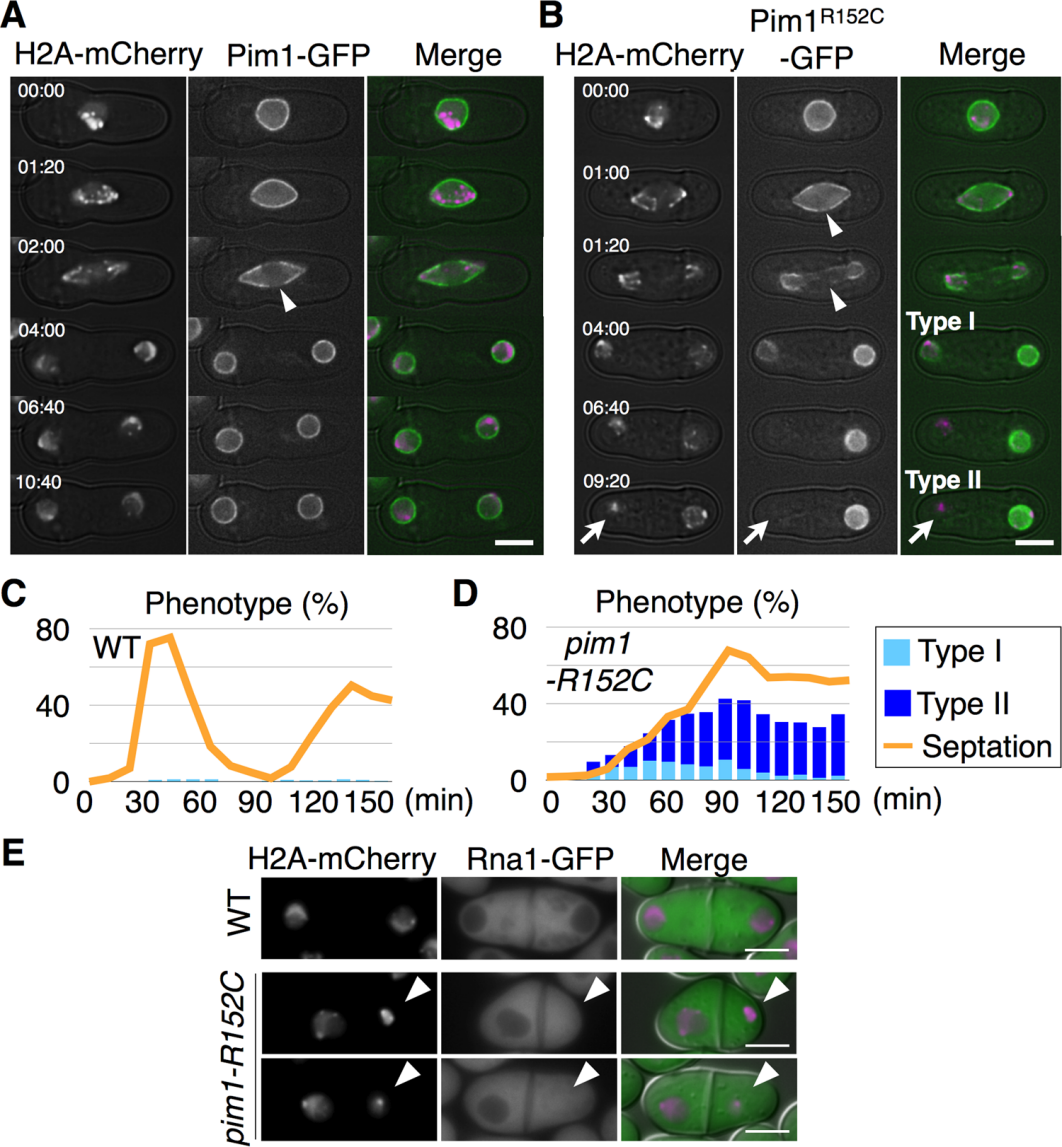
**The Ran-GTP gradient is collapsed in hypercondensed chromatin.** (A,B) Time-lapse observations of Pim1-GFP (*n*=10 cells) or Pim1^R152C^-GFP (*n*=10 cells) with H2A-mCherry were performed in YE (+Ade, Ura) medium at 20 s intervals. NIG8013 and NIG8015 were cultivated to log phase at 30°C and shifted to 37°C for 2 h. Green, Pim1-GFP; magenta, H2A-mCherry. Times are indicated in min:sec. (B) The arrows indicate H2A-mCherry signal hypercondensed at 09:20. The phenotype was observed in two of 10 cells. Type I: a binucleated cell with normal sized chromatin and decreased Pim1^R152C^-GFP signal. Type II: a binucleated cell with hypercondensed chromatin, without Pim1^R152C^-GFP signal. In both A and B, arrowheads indicate that the Pim1-GFP signal is decreased around the center of the nucleus during anaphase. (C,D) Synchronization experiments were performed once using NIG8013 and NIG8015, as shown in the Materials and Methods. (E) Rna1-GFP and H2A-mCherry were observed in the WT (*n*=274) and *pim1-R152C* (*n*=386). NIG8008 and NIG8009 were cultivated as in A and observed. Arrowheads indicate hypercondensed chromatin with Rna1-GFP. Green, Rna1-GFP; magenta, H2A-mCherry. Two independent experiments were performed. Scale bars: 5 µm.

To examine the localization of Pim1 in greater detail, a deconvolution method (Hiraoka et al., 1987), using GFP-fused Pim1, was performed. Fifty images of a cell were taken along the z-axis in 0.1 µm intervals by using a DeltaVision microscope and deconvolved. We found that the localization of Pim1-GFP showed dot-like signals on the nuclear envelope, and therefore presumed that Pim1-GFP was localized on the nuclear pore complex (Fig. 2A). Consistent with this result, the fluorescent intensity of Pim1-GFP or Pim1^R152C^-GFP decreased in the middle of the fusiform-shaped nuclear envelope, as shown by the arrowheads in Fig. 3A and B. This phenomenon was similar to the case of the nucleoporin Cut11-GFP (Aoki et al., 2011; Yam et al., 2011). Further, to confirm that Pim1 is localized on the nuclear pore complex, we performed an immunoprecipitation experiment. Strains harboring both Pim1-3Flag and Nup85-GFP grown at 37˚C for 2 h in YE (+Ade, Ura) medium were extracted and used for the reaction with an antibody against the nuclear pore complex, Mab414. We found that Pim1-3Flag was clearly immunoprecipitated with the nuclear pore complex (Fig. 2B).

Because it was previously reported that RCC1 homologs were mainly localized on chromosomes in several organisms (Ohtsubo et al., 1989; Lee et al., 1993; Matynia et al., 1996; Nemergut et al., 2001; Moore et al., 2002), we next examined whether the localization of Pim1 on the nuclear pore complex was dependent on the chromosomes in *S. japonicus*. For this purpose, we performed an *in situ* chromatin-binding assay with exponentially grown cells harboring both H2A-mCherry and Pim1-GFP at 30°C in YE (+Ade, Ura) medium. We found that the addition of DNase I to digest the DNA markedly reduced DAPI staining and H2A-mCherry levels, whereas the Pim1-GFP signal remained unaltered in 97.2% of the cells (Fig. 2C). Therefore, the localization of Pim1 on the nuclear pore complex did not depend on chromosomes in *S. japonicus*. Together, these results indicated that Pim1 was localized on the nuclear pore complex in a manner independent of the chromosomes.

### Mutated Pim1 was mislocalized on the nuclear pore complex

To examine the localization of mutated Pim1, an immunoprecipitation experiment with strains harboring both Pim1^R152C^-3Flag and Nup85-GFP grown at 37°C for 2 h in YE (+Ade, Ura) medium was performed. We found that Pim1^R152C^-3Flag interacted with the nuclear pore complex, but the level of interaction was 67.5% of that observed in the WT (Fig. 2B). Therefore, it was presumed that the interaction between Pim1 and the nuclear pore complex was weakened in *pim1-R152C*. Moreover, if the weakness of this interaction were the reason for the hypercondensation of chromatin, we would expect the phenotype to be suppressed by anchoring of a Pim1^R152C^ on the nuclear pore complex. To examine this possibility, we made a fusion construct between Pim1^R152C^ and a nucleoporin, and examined whether or not this fusion construct suppressed the phenotype of hypercondensed chromatin and the growth rate in *pim1-R152C*. As a nucleoporin, Ely5 was used (Asakawa et al., 2014). Both Pim1-Ely5-GFP and Pim1^R152C^-Ely5-GFP were localized on the nuclear envelope and the chromosome (Fig. S1B). We found that the phenotype of hypercondensed chromatin and the growth rate were suppressed by the expression of Pim1-Ely5, Pim1^R152C^-Ely5, and Pim1, but not by the expression of Pim1^R152C^ or Ely5 (Fig. 2D,E). These results suggested that the reduction in the interaction between Pim1 and the nuclear pore complex resulted in the production of the hypercondensed chromatin.

### Mislocalization of the mutated Pim1 occurred during the G1 phase and was followed by the hypercondensation of chromatin

To examine the cell cycle stage at which the hypercondensed chromatin appeared, we observed the localization of a mutated version of Pim1 throughout the cell cycle. Time-lapsed observations using cells harboring Pim1-GFP or Pim1^R152C^-GFP were performed 2 h after shifting the cells from 30°C to 37°C in YE (+Ade, Ura) medium. Pim1-GFP was mainly localized on the nuclear pore complex throughout the cell cycle, and it was equally divided between the daughter nuclei during mitosis (Fig. 3A). Similar to the case of Pim1-GFP, the main localization of Pim1^R152C^-GFP was on the nuclear pore complex, and its division during mitosis was equivalent between the daughter nuclei at 01:20 (Fig. 3B). However, the localization of Pim1^R152C^-GFP in one of the nuclei was diminished during the G1 phase, which finally led to the unequal localization of Pim1^R152C^-GFP in the binucleated cell at 09:20, as shown by the arrows in Fig. 3B. In addition, the reduced localization of Pim1^R152C^-GFP was followed by hypercondensation of chromatin (Fig. 3B). In the observation of Pim1^R152C^-GFP, we defined a binucleated cell having a normal-sized chromatin with decreased localization of Pim1^R152C^-GFP as type I, and a binucleated cell having a hypercondensed chromatin without localization of Pim1^R152C^-GFP as type II (Fig. 3B).

Confirmation of the phenotype was obtained from an experiment using synchronous cells harboring both Pim1^R152C^-GFP and H2A-mCherry (Fig. 3D). When synchronous cells were incubated at 37°C for 150 min, septated cells were accumulated in *pim1-R152C*, though their numbers oscillated in the WT (Fig. 3C and D). In concert with the accumulation of septated cells, the combined percentage of type I and type II cells reached ∼43% of the total cells at 90 min in *pim1-R152C* (Fig. 3D). In addition, the increase of type I cells occurred prior to that of type II cells in *pim1-R152C* (Fig. 3D). Moreover, this result was confirmed in a different set of synchronous cells harboring Pim1^R152C^-GFP and Cut11-mCherry (Fig. S2). Therefore, it was concluded that diminishment of Pim1^R152C^-GFP preceded the hypercondensation of chromatin. These results were consistent with the results from the time-lapsed imaging. Together, the findings indicated that chromosomes were hypercondensed during the G1 phase by a reduction in the localization of Pim1-GFP.

### The Ran-GTP gradient was lost in the hypercondensed chromatin

Our experiments thus suggested that the Ran-GTP gradient between the nucleus and cytoplasm was lost in the hypercondensed nucleus. To confirm this, we next examined the localization of Rna1, a homolog of RanGAP (Hopper et al., 1990), in *S. japonicus*, because the cytoplasmic localization of Rna1 showed that the Ran-GTP gradient was intact. We observed localization of GFP-fused Rna1 with H2A-mCherry 2 h after shifting the cells from 30°C to 37°C in YE (+Ade, Ura) medium. Rna1-GFP was localized in the cytoplasm but not in normal-sized nuclei, which created a gradient of Rna1-GFP across the nuclear envelope in the WT (Fig. 3E). However, the gradient disappeared in 86.4% of the hypercondensed chromatins (*n*=44) in *pim1-R152C*, as shown by the arrowheads in Fig. 3E. This result confirmed that the Ran-GTP gradient between the nucleus and cytoplasm was collapsed in the hypercondensed chromatin.

### Condensin was released from the chromosomes during the G1 phase in the WT

**Fig. 4.**
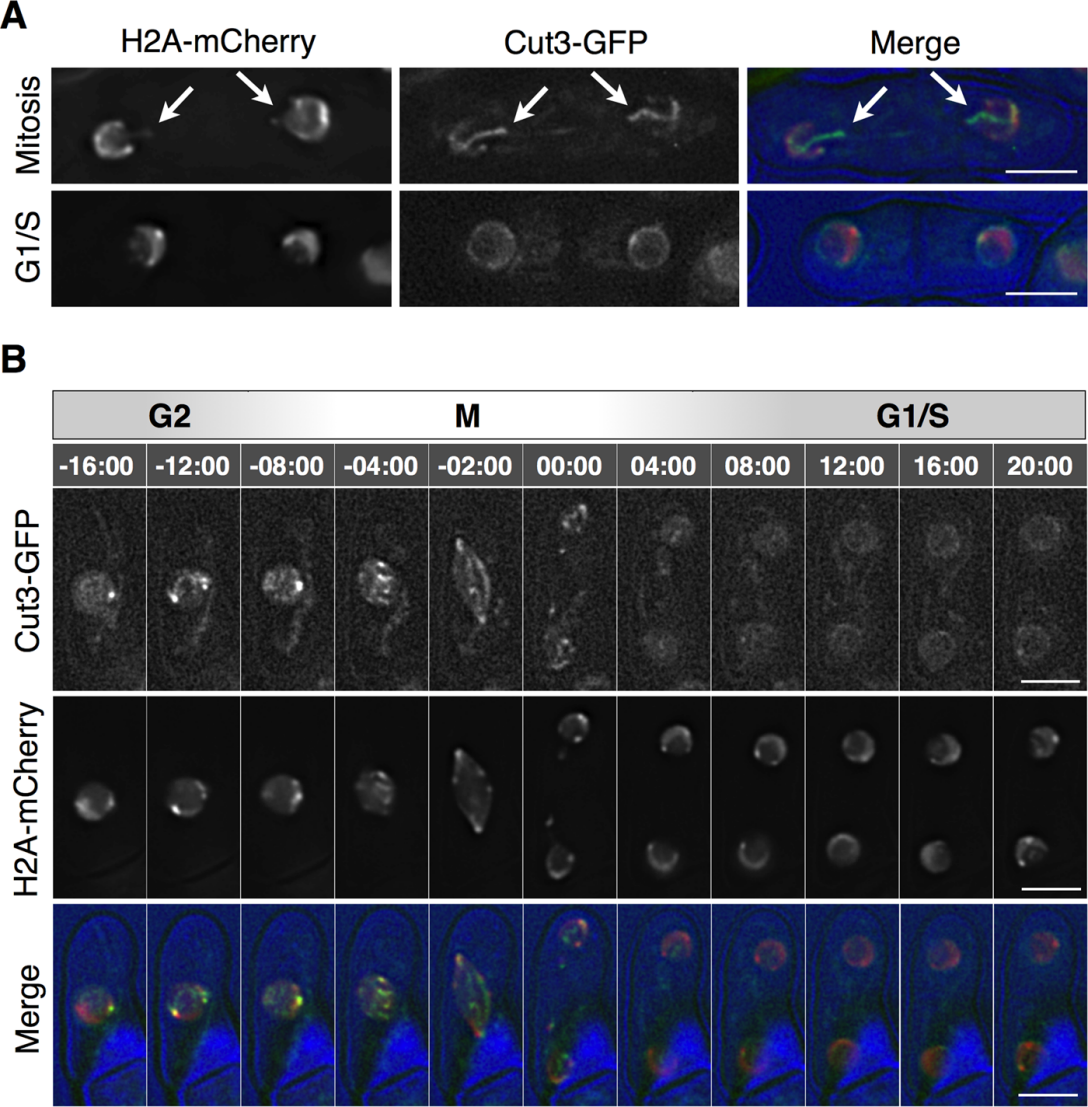
**Condensin is released from the chromosomes after mitosis in *S. japonicus*.** (A) Localization of Cut3-GFP (green) and H2A-mCherry (red) was observed in WT cells using a DeltaVision microscope (*n*=163). NIG8016 was cultivated in YE (+Ade, Ura) medium to log phase at 30°C, and shifted to 37°C for 2 h. Five images were taken at 0.5 µm intervals along the z-axis, then deconvolved. Blue, DIC images. Arrows indicate mitotic chromosomes. Two independent experiments were performed. (B) Time-lapse observations using NIG8016 was performed at 30°C in YE (+Ade, Ura) medium (*n*=11 cells). Times are indicated in min:sec. Scale bars: 5 µm.

How the collapse of the Ran-GTP caused the hypercondensation of chromatin has not been resolved; however, condensin would be expected to affect chromatin hypercondensation. Therefore, to examine the relationship between Ran-GTP collapse and the hypercondensation of chromatin, we first monitored the localization of Cut3, an SMC subunit of condensin, throughout the cell cycle in *S. japonicus*. From observations using cells harboring Cut3-GFP and H2A-mCherry at 37°C, we found that Cut3-GFP was localized on the three chromosomes during mitosis, as shown by the arrows in Fig. 4A, and it was localized on the nuclear envelope of a binucleated cell during the G1/S phase. In addition, the time-lapse observations showed that the localization of Cut3-GFP was dynamically changed between chromosomes during the G2 phase or mitosis, and the nuclear envelope during the G1/S phase. Cut3-GFP was not localized on the cytoplasm *in S. japonicus* (Fig. 4B). Similar results were also obtained from the observation of mCherry-fused Cut14, which is an SMC subunit of condensin (Fig. S3). The localization of condensin on the nuclear envelope was thus used as a marker of condensin release from the chromosomes in *S. japonicus*.

### Condensin was not released from the hypercondensed chromatin

**Fig. 5.**
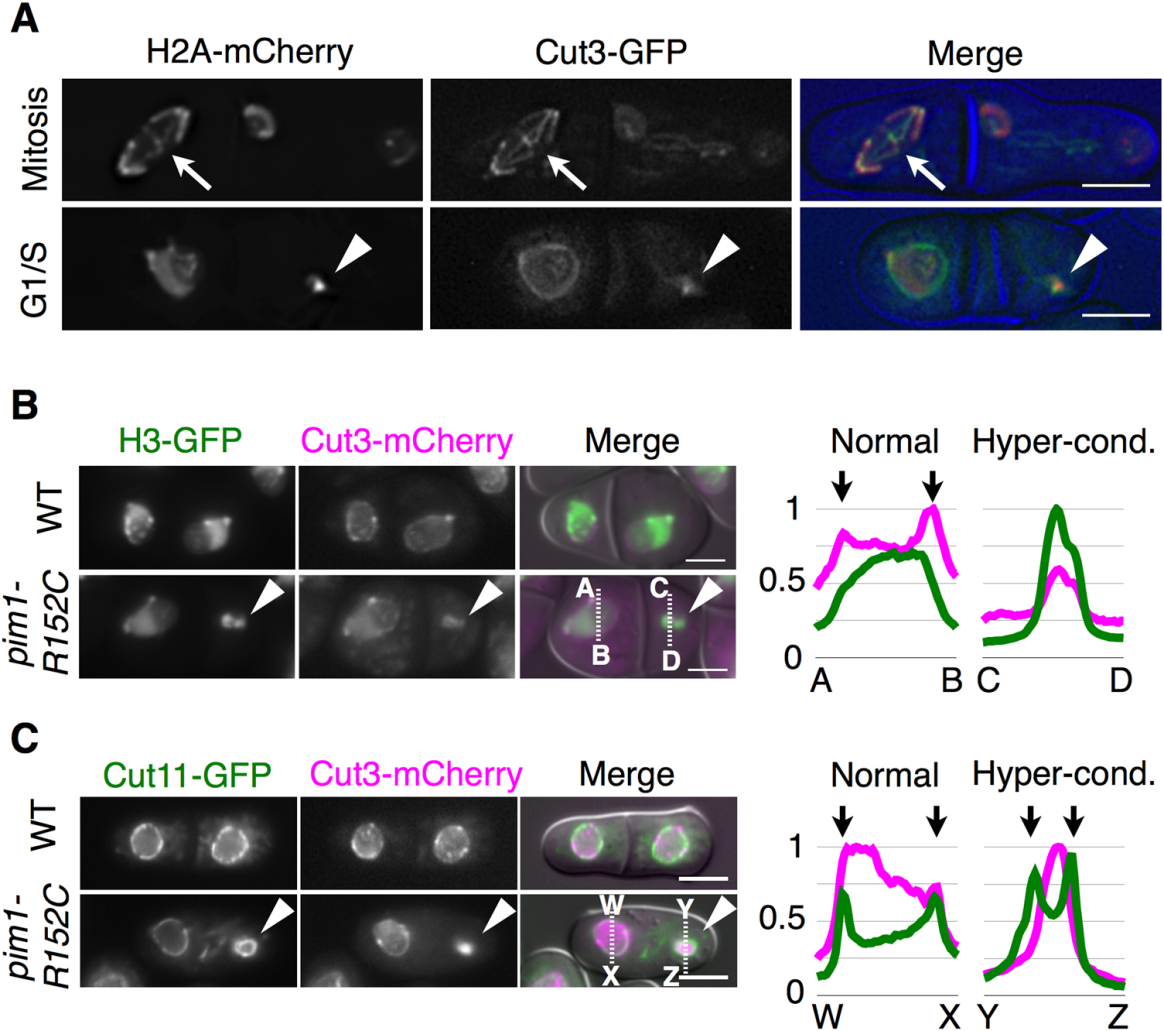
**Condensin is not released from hypercondensed chromatin.** (A) Localization of Cut3-GFP (green) and H2A-mCherry (red) was observed in *pim1-R152C* cells using a DeltaVision microscope (*n*=275). NIG8036 was cultivated in YE (+Ade, Ura) medium to log phase at 30°C, and shifted to 37°C for 2 h. Five images were taken at 0.5 µm intervals along the z-axis, then deconvolved. Blue, DIC images. Arrows indicate a mitotic chromosome. Arrowheads indicate hypercondensed chromatin. (B) Localization of Cut3-mCherry (magenta) and H3-GFP (green) was observed in *pim1-R152C* cells (*n*=1759) and WT cells (*n*=411). NIG8018 and NIG8020 were cultivated as in A and observed. Arrowheads indicate hypercondensed chromatin with Cut3-mCherry. Relative intensities of Cut3-mCherry and H3-GFP on the line of A-B or C-D were described by using ImageJ software (https://imagej.nih.gov/ij/). The length of the lines was 6.3 µm. Arrows indicate localization of Cut3-mCherry on the nuclear envelope. Green, H3-GFP; magenta, Cut3-mCherry. (C) Localization of Cut3-mCherry (magenta) and Cut11-GFP (green) was observed in *pim1-R152C* cells (*n*=550) and WT cells (*n*=375). NIG8021 and NIG8022 were cultivated as in A and observed. Arrowheads indicate hypercondensed chromatin with Cut3-mCherry. Relative intensities of Cut3-mCherry and Cut11-GFP on the line of W-X or Y-Z were described. The length of the lines was 5 µm. Arrows indicate the localization of Cut11-GFP on the nuclear envelope. Green, Cut11-GFP; magenta, Cut3-mCherry. Two independent experiments were performed in each analysis. Scale bars: 5 µm.

Next, to investigate whether or not condensin was released from the hypercondensed chromatin, we observed the localization of Cut3-GFP in *pim1-R152C*. Cells of *pim1-R152C* harboring Cut3-GFP with H2A-mCherry were observed 2 h after a temperature shift from 30°C to 37°C. As shown in Fig. 5A, we found that Cut3-GFP was localized on the three mitotic chromosomes (arrows), and was localized on the hypercondensed chromatin but not on the nuclear envelope during the G1/S phase (arrowheads). To confirm these results statistically, we observed 157 hypercondensed chromatins with Cut3-mCherry and H3-GFP, a fluorescently labeled version of the histone protein H3 used to visualize chromosomes, and found that 83.7% of the hypercondensed chromatins were colocalized with Cut3-mCherry signals, as shown by the arrowheads in Fig. 5B. Indeed, the relative intensity of Cut3-mCherry was merged with that of H3-GFP along the line of C-D on the hypercondensed chromatin. This was different from the case of normal-sized chromatin, in which the intensity of Cut3-mCherry was not merged with that of H3-GFP along the line of A-B because Cut3-mCherry was localized on the nuclear envelope, as shown by the arrows in Fig. 5B. In addition, we observed 76 hypercondensed chromatins with Cut3-mCherry and Cut11-GFP, and found that 85.6% of the condensed signals of Cut3-mCherry were surrounded by Cut11-GFP signals, as shown by the arrowheads in Fig. 5C. Indeed, the relative intensity of Cut3-mCherry was not merged with that of Cut11-GFP along the line of Y-Z in the hypercondensed chromatin. This was different from the case of normal-sized chromatin, in which the intensity of Cut3-mCherry was partially merged with that of Cut11-GFP along the line of W-X, as shown by the arrows in Fig. 5C. Therefore, the hypercondensed chromatin was accompanied by colocalization of condensin in *pim1-R152C*, suggesting that condensin was involved in the hypercondensation of chromatin.

### Association of condensin on the chromosomes was increased by a CDK phospho-mimic mutation

**Fig. 6.**
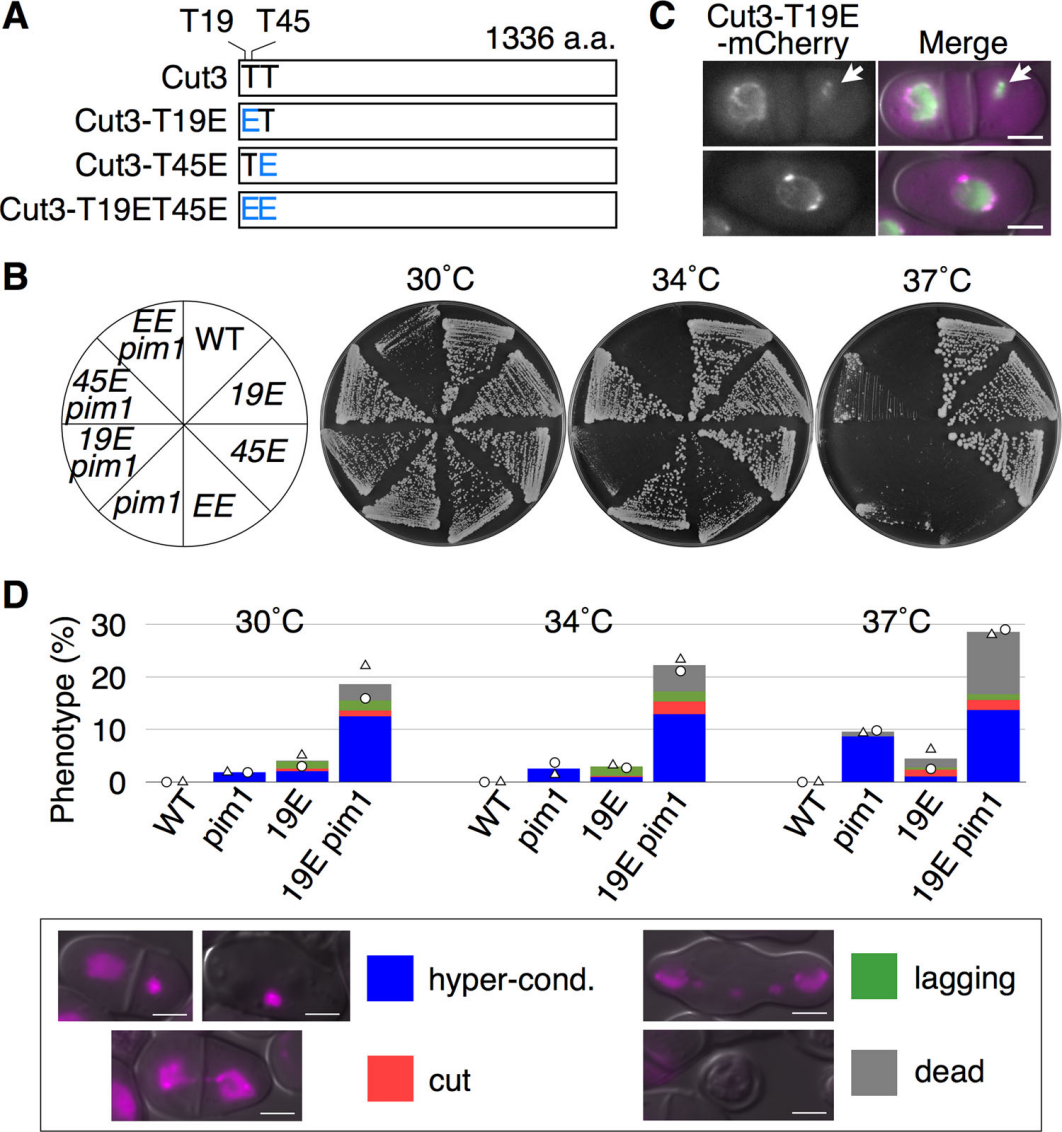
***pim1-R152C* was synthetic lethal with *cut3-T19E*.** (A) Threonine 19 and 45 in CDK phosphorylation consensus sites of Cut3 in *S. japonicus* were replaced by glutamate residues. (B) Growth rates of WT: NIG2028, *19E*: NIG8023, *45E*: NIG8024, *EE*: NIG8025, *pim1*: NIG8001, *19E pim1*: NIG8026, *45E pim1*: NIG8027 and *EE pim1*: NIG8028 were examined on YE (+Ade, Ura) plates at 30°C, 34°C and 37°C. (C) Localization of Cut3-T19E-mCherry (magenta) and H3-GFP (green) was observed in the hypercondensed chromatins (*n*=17). NIG8037 was cultivated in YE (+Ade, Ura) medium to log phase at 30°C. Arrows indicate hypercondensed chromatin. (D) Nuclear phenotypes were examined in NIG8003 (*n*=804 cells), NIG8004 (*n*=714 cells), NIG8029 (*n*=877 cells) and NIG8030 (*n*=804 cells). These strains were cultivated in YE (+Ade, Ura) medium to log phase at 30°C, shifted to 34°C or 37°C for 2 h then observed. Each symbol shows results from two independent experiments, and the bar graphs show the mean of these experiments. Each example of nuclear phenotypes stained by H2A-mCherry is shown. Two independent experiments were performed in each analysis. Scale bars: 5 µm.

To confirm that condensin was involved in the hypercondensation of chromatin, we examined whether or not the frequency of hypercondensed chromatin in *pim1-R152C* was increased by a phospho-mimic mutation of a CDK phosphorylation site of Cut3. In the protein sequence of Cut3 in *S. japonicus*, there were two CDK phosphorylation consensus sequences of ^19^TPDR and ^45^TPVR that corresponded with the authentic consensus sequence S/T-P-X-K/R (Hanks and Quinn, 1991). We replaced the 19th threonine or 45th threonine with glutamate to make two phospho-mimic mutants of Cut3: *cut3-T19E* and *cut3-T45E* (Fig. 6A). We compared the growth rates between the WT, *pim1-R152C*, the glutamate mutants and double mutants, and found that the growth rate of *cut3-T19E pim1-R152C* was synthetically lethal at 34°C and 37°C on YE (+Ade, Ura) plates (Fig. 6B). The localization of mCherry-fused Cut3-T19E was accumulated in the hypercondensed chromatins (82.4%), as shown by the arrows in Fig. 6C. Next, to examine the frequency of hypercondensed chromatin, localization of H2A-mCherry was observed in *cut3-T19E*, *cut3-T19E pim1-R152C* and *pim1-R152C*, with the WT as a control, at 30, 34 and 37°C for 2 h in YE (+Ade, Ura) medium*.* The frequency of the hypercondensed chromatin was increased in the *cut3-T19E pim1-R152C* in comparison with *cut3-T19E*, *pim1-R152C* and the WT (Fig. 6D). At 34°C, the frequency of the hypercondensed chromatin of *cut3-T19E pim1-R152C* was ∼14 times higher than that of *cut3-T19E*, and about five times higher than that of *pim1-R152C*. In addition, in *cut3-T19E pim1-R152C*, the accumulation of the phenotype of hypercondensed chromatin was about three times greater than the accumulations of the mitotic defective phenotypes, i.e. the *cut* phenotype and the *lagging* phenotype (Fig. 6D). Therefore, these results suggested that the hypercondensation of chromatin was increased by the CDK-phospho-mimic mutation in Cut3.

### Hypercondensed chromatin with localization of condensin was produced by deletion of a nucleoporin or addition of leptomycin B

**Fig. 7.**
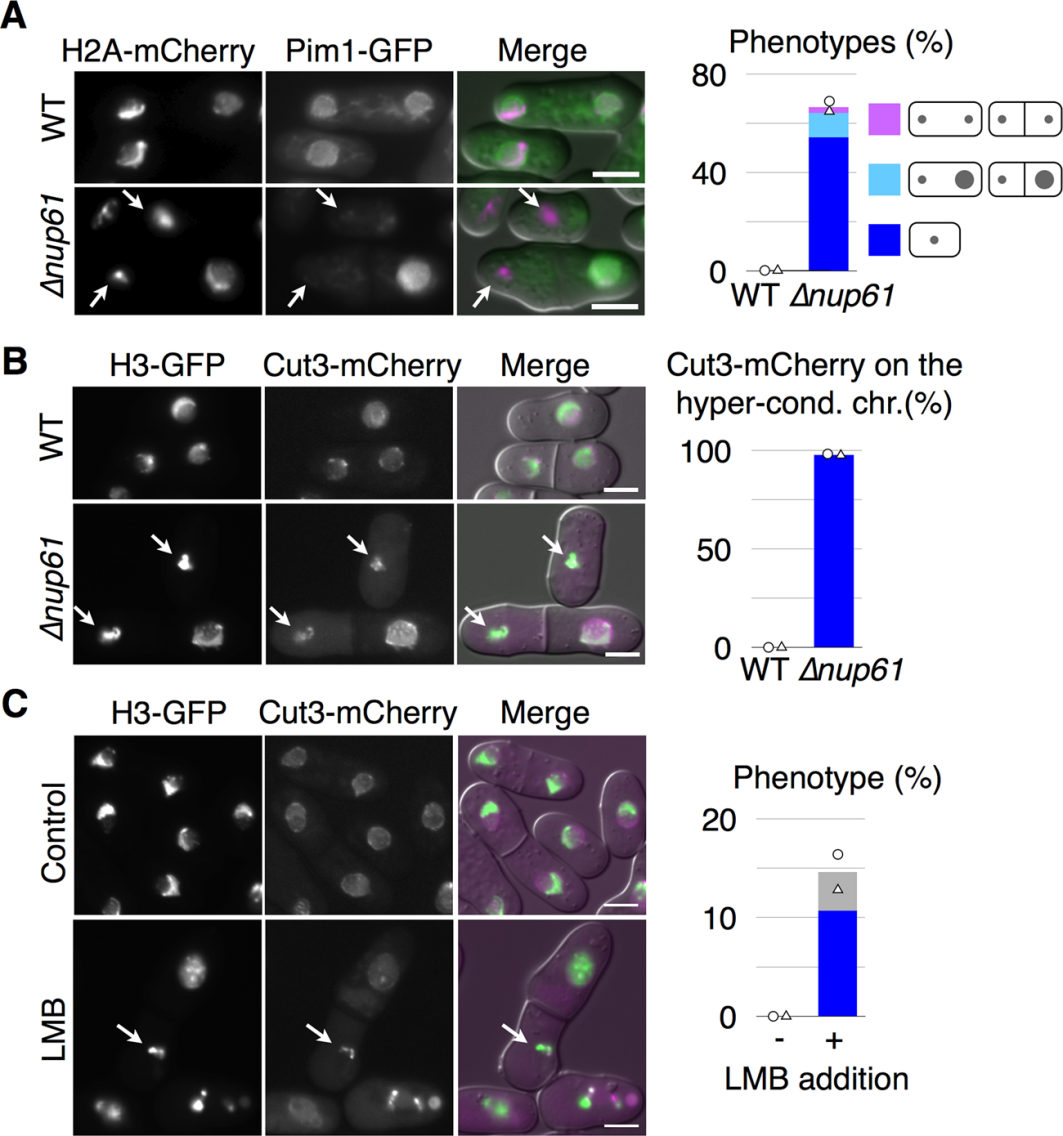
**Hypercondensed chromatin was produced by deletion of *nup61^+^* or addition of leptomycin B.** (A) Localization of Pim1-GFP (green) and H2A-mCherry (magenta) was observed in the Δ*nup61* mutant. NIG8013 and NIG8014 were cultivated to log phase in YE (+Ade, Ura) medium at 30°C and observed. Arrows indicate hypercondensed chromatin without localization of Pim1-GFP. The hypercondensed chromatin was observed in 66.7% of Δ*nup61* cells (*n*=209), but never observed in the WT (*n*=127). (B) Localization of Cut3-mCherry (magenta) and H3-GFP (green) was observed in the Δ*nup61* cells (*n*=394) and WT cells (*n*=529). NIG8018 and NIG8019 were cultivated as in A, and 97.9% of the hypercondensed chromatins (*n*=96 cells) were colocalized with signals of Cut3-mCherry in Δ*nup61*, as shown by the arrows. (C) Localization of Cut3-mCherry (magenta) and H3-GFP (green) was observed in leptomycin B (LMB)-treated cells. NIG8018 was cultivated as in A and treated with 4 µM LMB or ethanol (control). After the treatment, the cells were cultivated at 30°C for 4 h and observed. In the treated cells, 14.6% of cells (*n*=539) showed hypercondensed chromatin. In addition, 73.3% of the hypercondensed chromatins (blue) was colocalized with signals of Cut3-mCherry, as indicated by the arrows. In A, B and C, each symbol shows results from two independent experiments, and the bar graphs show the mean of these experiments. Two independent experiments were performed in each analysis. Scale bars: 5 µm.

The above results suggested that the release of condensin from chromosomes might be dependent on the interaction between Pim1 and the nuclear pore complex. If this were the case, the localizations of Pim1-GFP and Cut3-mCherry would collapse in a mutant of the nuclear pore complex. To examine this possibility, we artificially disturbed the nuclear pore complex in a manner independent of Pim1 function. We developed a gene-disrupted mutant of *nup61^+^* that was an ortholog of a nucleoporin Nup50 in higher eukaryotes (Guan et al., 2000). From the observation of the Δ*nup61* harboring both H2A-mCherry and Pim1-GFP at 30°C in YE (+Ade, Ura) medium, we found that the hypercondensed chromatin was accumulated in 66.7% of the cells (Fig. 7A). In addition, Pim1-GFP was not localized on the hypercondensed chromatin, as shown by the arrows in Fig. 7A. It was noted that the phenotype of Δ*nup61* was more severe than that of *pim1-R152C*. Moreover, based on another observation using the Δ*nup61* harboring both H3-GFP and Cut3-mCherry, we found that Cut3-mCherry was persistently accumulated on 97.9% of the hypercondensed chromatin, as shown by the arrows in Fig. 7B. These results confirmed that the nuclear pore complex was required in order to achieve adequate localizations of Pim1 and condensin in the nucleus. In addition, we also examined whether the localization of condensin was disturbed by treatment with leptomycin B, which is an inhibitor of Crm1 (Kudo et al., 1999). Exponentially grown cells harboring H3-GFP and Cut3-mCherry at 30°C in YE (+Ade, Ura) medium were treated with leptomycin B and observed under a microscope. We found that 14.6% of the treated cells showed hypercondensed chromatin, and Cut3-mCherry was colocalized with the hypercondensed chromatin in 73.3% of these cells (Fig. 7C). The hypercondensed chromatins that were obtained by treatment with leptomycin B were surrounded by both Cut11-GFP and GFP-AHDL, but they did not exhibit exclusion of Rna1-GFP and localization of Pim1-GFP or mCherry-NLS (Fig. S4). From these results, it was suggested that active exclusion of condensin or some condensation factors from the chromosomes is required for the chromosome decondensation.

## DISCUSSION

In this paper, we have shown that Pim1 plays an important role in the dissociation of condensin from mitotic chromosomes in *S. japonicus*. Our major observations are as follows: (i) a temperature-sensitive mutant, *pim1-R152C*, showed hypercondensed chromatin in *S. japonicas*; (ii) Pim1 was localized on the nucleus by anchoring on the nuclear pore complex, and a failure of this localization resulted in hypercondensation of chromatin; (iii) the hypercondensed chromatin was accompanied by colocalization of condensin; (iv) hypercondensed chromatin in the *pim1-R152C* was increased by a CDK phospho-mimic mutation of Cut3.

**Fig. 8.**
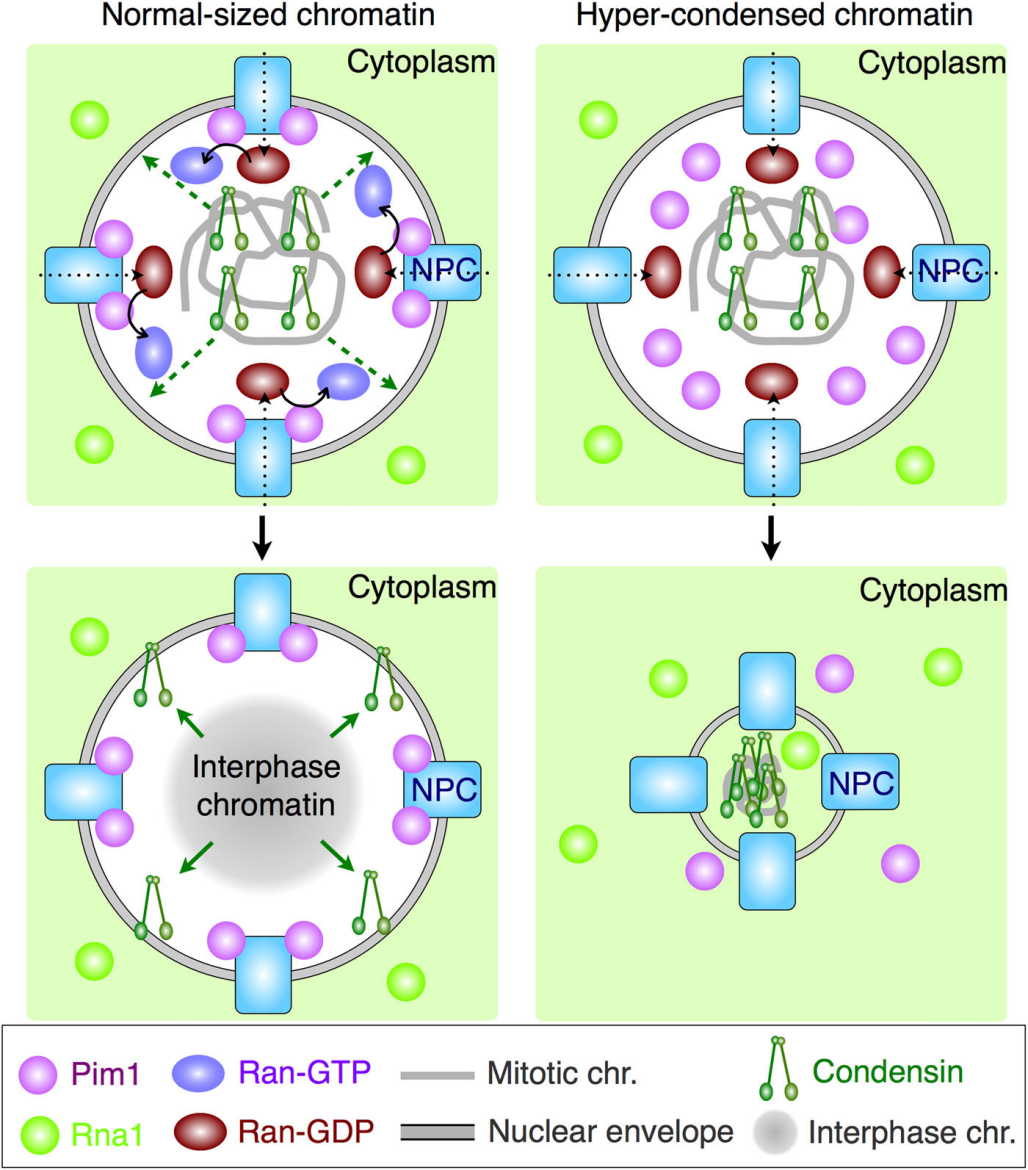
**Schematic of the model investigated in this study.** Condensin is dissociated from chromosomes in a Ran-GTP-dependent manner during the G1 phase. NPC, nuclear pore complex.

Pim1-GFP was mainly localized on the nuclear pore complex throughout the cell cycle in the WT. Pim1 would be imported into the nucleus by the NLS (Seino et al., 1992), then either localize on the nuclear pore complex or on chromosomes. However, the localized Pim1^R152C^-GFP disappeared from the nuclear pore complex during G1 phase, which was followed by chromatin hypercondensation. We assume that the conserved 152nd arginine in Pim1 is required for its localization on the nuclear pore complex or its level during G1 phase. In addition, the 152nd arginine corresponds to the 142nd arginine of *S. pombe* Pim1, which exists on the Ran-interacting domain of the second RCC1-blade (Hirose et al., 2006). Therefore, the 152nd arginine would be simultaneously important for both the localization ability and interaction with Ran. The Pim1^R152C^ would retain the WT level of Ran nucleotide exchange activity at 37°C, because the fusion of Pim1^R152C^-Ely5 suppressed the hypercondensed chromatin in *pim1-R152C*. If Pim1 were not localized on the nuclear pore complex, the Ran-GTP gradient would not be well established (Fig. 8).

The association of Pim1/RCC1 homologs on the nuclear pore complex was previously reported. In that report, the nucleotide exchange on Ran occurred at the nuclear pore complex (Fontoura et al., 2000). In addition, another report indicated that Ran interacted with the nuclear pore complex (Saitoh et al., 1996; Bai et al., 2004). On the other hand, Pim1/RCC1 homologs are known to be localized on the chromosomes and to form a Ran-GTP gradient around the chromosomes (Kalab et al., 2002). Based on these findings, we conjecture that the localization of Pim1/RCC1 on both the nuclear pore complex and the chromosomes is conserved among species. The proportion of Pim1/RCC1 localized at each of these sites would differ among species. The association of Pim1/RCC1 on the nuclear pore complex would be more unstable or dynamic than that on the chromosomes, because the Pim1/RCC1 is abundantly localized on the chromosomes in most species. Further investigations will be needed to understand why Pim1 tends to be so highly localized on the nuclear pore complex in *S. japonicus*.

Our results suggest that chromatin hypercondensation is a consequence of the condensin being located on chromosomes even after mitosis. Two experiments supported this suggestion. First, we found that condensin was dissociated from the chromosomes and localized on the nuclear envelope during the G1 phase in the WT. However, this dissociation did not occur on the hypercondensed chromatin in *pim1-R152C*. The condensed signals of condensin were colocalized with signals of H2A-mCherry but not with signals of Cut11-GFP. Pim1 was required to dissociate condensin from mitotic chromosomes. Condensin would be released from chromosomes in a Ran-GTP-dependent manner. Second, *pim1-R152C* was shown to be synthetic lethal with *cut3-T19E*. The frequency of the hypercondensed chromatin was increased, and the growth rate was decreased, in the double mutant of *pim1-R152C cut3-T19E*, but not in *pim1-R152C cut3-T45E*. This indicated that the phospho-mimic mutation of 19th threonine, but not 45th threonine, in Cut3 inhibited its release from chromosomes. The phosphorylation of the 19th threonine in Cut3 would be required for condensin localization on the chromosomes in *S. japonicus*. Previous reports indicated that condensin is activated by CDK phosphorylation (Kimura et al., 1998, 1999). Therefore, the hypercondensation of chromatin would be caused by the untimely localization of the active condensin. Further, it might be presumed that the inactive condensin suppresses the hypercondensation of chromatin in *pim1-R152C*. In addition to the phosphorylated 19th threonine in Cut3, other phosphorylated residues in condensin might have to be dephosphorylated for the condensin release, because the phenotype of the hypercondensed chromatin in the double mutant of *cut3-T19E pim1-R152C* was partial. Not only other potential CDK sites but also Aurora sites (Giet and Glover, 2001; Takemoto et al., 2007; Nakazawa et al., 2011) or Polo sites (St-Pierre et al., 2009) would be candidates, because their phosphorylation activates condensin. Alternatively, we cannot exclude the possibility that a compaction of the nuclear envelope causes the hypercondensation of chromatin. This is because it is difficult to distinguish between the hypercondensation of chromatin and the compaction of the nuclear envelope due to chromosomes interacting with the nuclear envelope (Mekhail and Moazed, 2010). If the hypercondensation of chromatin was solely due to the compaction of the nuclear envelope, we would expect condensin to be dissociated from the hypercondensed chromatin and localized on the nuclear envelope in *S. japonicus*. However, our results indicated that condensin remained on the hypercondensed chromatin. Therefore, these results suggested that the hypercondensation of chromatin was not caused by compaction of the nuclear envelope alone.

The collapse of the Ran-GTP gradient would cause the mislocalization of several nuclear proteins that regulate the release of condensin. In terms of the mechanism by which proteins regulate condensin release, we propose the following. First, it may be that a phosphatase for condensin is mislocalized by the collapse of the Ran-GTP gradient, resulting in a failure of the dephosphorylation of condensin. Type 1 protein phosphatase or Cdc14 phosphatase would be candidates for the phosphatase for condensin, because they are localized in the nucleus (Fernandez et al., 1992; Andreassen et al., 1998; Stegmeier and Amon, 2004) and they function in the mitotic exit. Malfunction of these enzymes is known to cause a mitotic defect (Ohkura et al., 1989; Fernandez et al., 1992; Stegmeier and Amon, 2004). Condensin might be dephosphorylated in a manner dependent on the PP1-PP2A phosphatase relay (Grallert et al., 2015) or SIN pathway (Simanis, 2003). In addition, Ptn1 (Mitra et al., 2004), which is a PTEN ortholog, would also be a candidate for the phosphatase for condensin, because Ptn1 is partially localized on the nucleus in binucleated cells in *S. pombe* (Mitra et al., 2004), and it has been described that PTEN is localized in the nucleus in a Ran-GTP-dependent manner in humans (Gil et al., 2006). A second possibility may be that Ran-GTP regulates the activity of the phosphatase indirectly. This possibility was suggested by a previous report that the Ran pathway regulates the phosphorylation level of Eg2 through TPX2 in microtubule regulation (Tsai et al., 2003). A third possibility may be that nuclear import or export of some regulators for condensin is important for condensin remodeling, so that disrupting the compartmental identity of the nucleus in *pim1-R152C* would block the condensin release. A fourth possibility may be that the condensin release from the mitotic chromosomes is dependent on the spindle pole body, because the condensed chromatin is associated with the newer spindle pole body in the *pim1-d1* mutant in *S. pombe* (Gonzalez et al., 2009). In any case, after condensin is dissociated from the chromosomes, the chromosomes are decondensed. In the event that condensin is not dissociated from the chromosomes, the chromosomes would become hypercondensed (Fig. 8). Based on this model, we argue that the loss of Ran-GTP causes a postmitotic condensation of chromatin by condensin during the G1 phase. From this point of view, it becomes clear why hypercondensed chromatin is produced by the mutations of RCC1 homologs in other organisms. It was previously suggested that GTPase was involved in decondensation of chromatin (Sazer and Nurse, 1994; Magalska et al., 2014). Our present results further suggest a possible mechanism by which GTPase affects chromosome decondensation.

However, the model does not clarify why the mitotic chromosomes are not hypercondensed. We hypothesized that there may be an unknown decondensation factor in the nucleus, and this factor could inhibit the hypercondensation of the mitotic chromosomes. Further, the activity or localization of such a decondensation factor could depend on Ran-GTP. We consider that the discrepancy between mitotic chromosomes and hypercondensed chromosomes likely indicates the presence of an unknown decondensation factor.

In conclusion, we found that Pim1/RCC1 was required for the dissociation of condensin from mitotic chromosomes in *S. japonicus*. This is the first report to describe the combined role of Pim1/RCC1 and the condensin dynamics in regulating chromosome decondensation.

## MATERIALS AND METHODS

### Strains and media

All strains used in this study are listed in Table S1. Detailed information on the plasmid used to construct the strains is presented under ‘Plasmid construction’. Haploid strains were derivatives of NIG2028 or NIG2017 (Furuya and Niki, 2009). Cells were cultivated in YE medium (0.5% yeast extract, 3% glucose) supplemented with 100 µg/ml adenine and 100 µg/ml uracil (Furuya and Niki, 2009). When cells were transformed by a pSJU11-based multi-copy plasmid (Aoki et al., 2010), EMM2 plates were used as a selective medium. When cells were transformed by a pFA6a-based plasmid (Bahler et al., 1998), YE (+Ade, Ura) plates with 40 µg/ml G418 (Alexis, Enzo, NY) or 40 µg/ml clonNAT (HKI, Jena) were used as a selective medium. Transformation was performed using the electroporation method (Aoki et al., 2010). Agar plates included 2% agar. To monitor the growth of cells, the turbidity of cell cultures was measured using a Klett-Summerson colorimeter (Thomas Scientific, Swedesboro, USA) (Aoki et al., 2010). EMM2 was composed of 2.2 g Na_2_HPO_4_, 3.0 g potassium hydrogen phthalate, 5.0 g NH_4_Cl, 20 g D-glucose, salt stock (1×), vitamin stock (1×), mineral stock (1×), and 100 mg/ml each of arginine, adenine, glutamic acid, leucine, lysine, and histidine per liter. The salt stock (50×) was composed of 53.5 g MgCl_2_-6H_2_O, 0.74 g CaCl_2_-2H_2_O, 50 g KCl, and 2 g Na_2_SO_4_ per liter. The vitamin stock (1000×) was composed of 1 g sodium pantothenate, 10 g nicotinic acid, 1 g inositol, and 10 mg biotin per liter. The mineral stock (10,000×) was composed of 5 g H_3_BO_3_, 4 g MnSO_4_, 4 g ZnSO_4_-7H_2_O, 2 g FeCl_3_-6H_2_O, 0.4 g H_2_MoO_4_-H_2_O, 1.0 g KI, 0.4 g CuSO_4_-5H_2_O, and 10 g citric acid per liter.

### Genomic sequence data of *S. japonicus*

Genomic sequence data of *S. japonicus* were referenced from the genomic database of the National Center for Biotechnology Information (NCBI) server (http://www.ncbi.nlm.nih.gov/) (Rhind et al., 2011). We newly used the sequences of *pim1^+^* (SJAG_04464.5), *rna1^+^* (SJAG_04400.5), *lem2^+^* (SJAG_01745.5), *nup85^+^* (SJAG_00471.5), *ely5^+^* (SJAG_01833.5), *nup61^+^* (SJAG_04284.5), and *cut3^+^* (SJAG_00871.5), which were annotated in the server.

### Isolation of a *pim1* mutant

The ts581 was isolated from a temperature-sensitive mutant library (Aoki et al., 2013). The ts581 was back-crossed three times with the WT strain before use. To determine mutations in the genome of ts581, the whole genome of ts581 was extracted using a Wizard Genomic DNA Purification Kit (Promega, Madison, USA) and sequenced at the TAKARA Dragon Genomics Center. To determine the causative genes, the genetic distances were checked by octad analyses involving the *ts* mutants, and a tester strain had an insertion of a drug-resistant gene near the causative gene, by the SINGER MSM system. The formula used to determine genetic distance was: cM=100×(TT+6NPD)/2(PD+NPD+TT) (Furuya and Niki, 2009). The genetic distance between ts581 and the *pim1* tester strain was within 1.3 cM (PD:TT:NPD=39:0:0).

### Observation of the growth rate and viability

The cells were exponentially grown in YE (+Ade, Ura) medium at 30°C and transferred to 37°C for 6 h in a water bath shaker in Fig. 1C and D. The volume of each cell culture was 50 ml in a glass flask. To determine growth rates, cell density at each time point was examined by a counting chamber (Hirschmann, Eberstadt, Germany) under the microscope every 2 h. To determine cell viabilities, a drop of culture containing 300 cells based on the cell density was added to each of two plates containing YE (+Ade, Ura) for incubation at each time point. From the numbers of cells grown after 2 days, we calculated the ratio of cell viability. To observe the nuclear phenotypes, each 1 ml sample was transferred to a 1.5 ml tube and concentrated 10 times in YE (+Ade, Ura) medium by centrifugation of 800 ***g*** for 2 min at room temperature. The samples were observed immediately under an AxioVision microscope (Zeiss, Oberkochen, Germany) with a Zeiss objective lens (63×).

### Microscopic analysis

To observe the nuclear phenotypes, cells were exponentially grown in YE (+Ade, Ura) medium at 30°C, and incubated for 2 h after a temperature shift from 30°C to 37°C in a water bath shaker before observation. The volume of each cell culture was 5 ml in a 50 ml Corning tube. After the incubation at 37°C, 1 ml of each cell culture was transferred to a 1.5 ml tube and concentrated 10 times in YE (+Ade, Ura) medium by centrifugation at 800 ***g***, for 2 min at room temperature. The samples were observed immediately under an AxioVision microscope with a Zeiss objective lens (63×). The cells in this experiment were not fixed.

### Time-lapse imaging

For the time-lapse imaging, a DeltaVision microscope (GE Healthcare, Boston, USA) with a 100× objective lens (Olympus, Tokyo, Japan) was used. Cells were cultivated to log phase in YE (+Ade, +Ura) medium at 30°C, and incubated for 2 h after a temperature shift from 30°C to 37°C in a water bath shaker before observation. 120 µl of cell culture was transferred to a glass-bottomed dish (Matsunami, Osaka, Japan). The living cells were observed every 20 s using the DeltaVision microscope, as previously described (Aoki et al., 2013). Image quality was enhanced by the deconvolution method using softWoRx (www.appliedp.com/en/default.htm) software (Applied Precision).

### Synchronization of the cells

To gather cells that were synchronized at the same stage of the cell cycle, elutriation was performed according to a modified version of the traditional method for *S. pombe* using a JE-5.0 elutriation rotor (Beckman Coulter, California, USA) (Aoki et al., 2011; Aves et al., 1985). *S. japonicus* cells were harvested following cultivation to log phase in 2 L of YE (+Ade, Ura) medium at 30°C. The cells were suspended in 10 ml of YE (+Ade, Ura) medium and sonicated three times for 10 s each by a Bioruptor (Cosmo Bio, Tokyo, Japan). The cells were elutriated, and small cells in the early G2 phase were collected. The small cells were re-incubated in YE (+Ade, Ura) medium at 37°C for 150 min. Subsequently, 5 ml of cell culture was collected every 10 min, then fixed with 3% paraformaldehyde and 0.25% glutaraldehyde diluted in PEM buffer and incubated for 1 h. The cells were harvested and washed twice with PEM buffer before observation. The PEM buffer consisted of 100 mM PIPES, 1 mM EGTA and 1 mM MgSO_4_, pH 6.9. The numbers of cells analyzed at each time point in Fig. 3C and D were as follows: in the WT, 0 min (*n*=149), 10 min (*n*=162), 20 min (*n*=229), 30 min (*n*=297), 40 min (*n*=269), 50 min (*n*=288), 60 min (*n*=244), 70 min (*n*=315), 80 min (*n*=203), 90 min (*n*=223), 100 min (*n*=207), 110 min (*n*=173), 120 min (*n*=196), 130 min (*n*=250), 140 min (*n*=379), 150 min (*n*=235) (Fig. 3C); in *pim1-R152C*, 0 min (*n*=303), 10 min (*n*=215), 20 min (*n*=382), 30 min (*n*=178), 40 min (*n*=327), 50 min (*n*=284), 60 min (*n*=252), 70 min (*n*=372), 80 min (*n*=307), 90 min (*n*=253), 100 min (*n*=240), 110 min (*n*=226), 120 min (*n*=386), 130 min (*n*=333), 140 min (*n*=256), 150 min (*n*=285) (Fig. 3D).

### Cell extract preparation

For immunoprecipitation experiments, 50 ml of exponentially growing cells were washed once with PBS buffer and then suspended in lysis buffer (50 mM Tris-HCl, pH 7.5, 150 mM NaCl, 1 mM EDTA, 1% NP-40, 10% glycerol, protease inhibitor cocktail tablets (Roche, Basel, Switzerland), 50 µM MG132, 1 mM PMSF). To disrupt the cells, the suspension was smashed at 3000 rpm for 30 s using a Micro Smash MS100 ball mill homogenizer (TOMY, Tokyo, Japan) with zirconia balls (Nikkato Corp., Osaka, Japan) at 4°C. The resulting suspension was centrifuged at 800 ***g*** for 5 min to collect the supernatant of the crude cellular extract at 4°C. The supernatant was diluted in dilution buffer (10 mM Tris-HCl, 150 mM NaCl, 0.5 mM EDTA) to produce a final volume of 0.55 ml. Then, 50 µl of the diluted suspension was used as an ‘input’ sample, and 0.5 ml of the diluted suspension was incubated for 30 min at 4°C by a rotator with IgG-treated protein G dynabeads (VERITAS, Tokyo, Japan) for pre-absorption. The supernatant after pre-absorption was incubated for 1 h at 4°C by a rotator with antibody-treated protein G dynabeads for immunoprecipitation. The dynabeads were collected on a magnetic stand and washed three times for 10 min each with the dilution buffer at 4°C. The dynabeads were then collected and treated with 50 µl SDS sample buffer (62.5 mM Tris-HCl, 1% SDS, 10% glycerol, 0.025% v/v mercaptoethanol, 0.001% v/v Bromophenol Blue) and boiled for 5 min. The resulting suspension was centrifuged at 15,300 ***g*** for 5 min at 4°C to collect the supernatant. The supernatant was used as a ‘pellet’ (Ppt) sample for blotting analysis. The immunoprecipitated proteins in the pellet sample were concentrated to a density 10-fold greater than that in the input sample. An antibody used for protein immunoprecipitation was the anti-nuclear pore complex protein antibody Mab414 (Abcam). For immunoprecipitation, 5 µl (1 mg/ml) of the Mab414 and 50 µl (30 mg/ml) of protein G dynabeads were used for a sample. For pre-absorption, 5 µl (1 mg/ml) of a mouse IgG1 isotype control (MBL, Nagoya, Japan) and 50 µl (30 mg/ml) of protein G dynabeads were used for a sample. Proteins in the sample were separated by electrophoresis on a 12% acrylamide gel. For protein blotting, Protran Premium 0.45 µm NC (Amersham, Buckinghamshire, UK) was used. Flag M2 antibody (1/500 dilution; Sigma-Aldrich), GFP antibody (1/500 dilution; Roche), and tubulin antibody (1/50,000 dilution; Sigma-Aldrich) were used to detect proteins. Western lightning plus-ECL (PerkinElmer, Waltham, USA) was used as a chemiluminescence reagent.

### *In situ* chromatin binding assay

*In situ* chromatin binding assays were performed as described previously (Kearsey et al., 2000) with minor modifications. First, 50 ml of exponentially grown cells in YE (+Ade, Ura) medium were harvested and washed once with ZM buffer (50 mM sodium citrate, pH 5.6, 1.2 M sorbitol, 0.5 mM MgAc, 10 mM DTT). The cells were resuspended in ZM buffer with 2 mg/ml zymolyase (Nacalai Tesque, Kyoto, Japan) and incubated for 1 h at 32°C. The cell suspension was then mixed with STOP buffer (100 mM MES, pH 6.4, 1.2 M sorbitol, 1 mM EDTA, 0.5 mM MgAc) and washed twice with STOP buffer. Next, the cell suspension was washed with EB buffer (20 mM PIPES-KOH, pH 6.8, 400 mM sorbitol, 2 mM MgAc, 150 mM KAc) and resuspended in EB2 buffer (20 mM Pipes-KOH, pH 6.8, 400 mM sorbitol, 5 mM MgAc, 150 mM KAc, 0.001% v/v of protease inhibitor cocktail, and 1% Triton X-100) and incubated for 7 min at 20°C. Half of the cell suspension was mixed with 0.1% v/v of 1 mg/ml DNaseI (Boehringer, Ingelheim, Germany) and the other half was mixed with water as a control. Both halves of the cell suspension were then incubated for 30 min at 0°C and mixed with NaCl to a final concentration of 250 mM. The cell suspensions were spun down, the supernatant was removed, and the cell pellets were washed once with methanol and once with acetone. Finally, the cell pellets were suspended with PBS buffer containing 0.4 µg/ml DAPI before observation. The cells were observed under an AxioVision microscope with a Zeiss objective lens (63×).

### Plasmid construction

Epitope tagging by GFP, mCherry or Flag was performed in the C-terminal regions of each protein. Locations within 0.5 kbp of the C-terminal region of a gene and 0.5 kbp of the 3′ untranslated region of a gene were amplified from the *S. japonicus* genome by polymerase chain reaction (PCR) and cloned into the BamHI (or SmaI)-AscI (or SmaI) and PmeI-EcoRI sites of pFA6aKanMX or pFA6aNatMX, which contain the sequences for GFP, mCherry or Flag, respectively (Bahler et al., 1998). The constructions of Cut11-GFP, H2A-mCherry and H3-GFP were previously reported (Aoki et al., 2011, 2013; Furuya and Niki, 2010). The multi-copy plasmid pSJU11 contains an autonomously replicating sequence and a *spura4^+^* sequence was used to clone the DNA fragments (Aoki et al., 2010). To purify plasmid DNA, the alkali method was used for small-scale plasmid preparation, and a JETstar 2.0 plasmid purification kit (VERITAS) was used for large-scale plasmid preparation.

To generate multi-copy plasmid of *pim1^+^*, the DNA fragments containing *pim1^+^* were amplified between the sequences of NotI-GTAGAATCCTAACGAGCGACAAAC and NotI-CTGTCAAAGGCGTTGACAACGTTC from purified genomic DNA by PCR and inserted into the NotI site of pSJU11.

To generate 3Flag-fused Pim1 (SJAG_04464.5), two DNA fragments were amplified from NIG2017 genomic DNA using the primer pairs BamHI-GTTGAGTCTATTACCGGTGGTGAG and SmaI-ATTAGACGTGTAAACTGTTTCTGT (Pim1-fragment 1), and PmeI-TTTCTTGCTGCGAATTGCCAATTT and EcoRI-CTGTCAAAGGCGTTGACAACGTTC (Pim1-fragment 2). Pim1-fragments 1 and 2 were inserted into the BamHI-SmaI and PmeI-EcoRI sites, respectively, of pFA6aNat-3Flag (a modified version of plasmid pFA6aNat-3Flag; provided by Dr Shigeaki Saitoh and Dr. Kohta Takahashi, Kurume University, Japan). The resulting plasmid, pPim1-3Flag-Nat, was digested with BamHI and EcoRI, and then transformed into NIG2028 using electroporation. To generate GFP-fused Pim1, a DNA fragment was amplified from NIG2017 genomic DNA using the primer pairs BamHI-GTTGAGTCTATTACCGGTGGTGAG and TGAAAAGTTCTTCTCCTTTACTTCCTCCTCCATTAGACGTGTAAACTGTTTCT (Pim1-fragment 3). In addition, a DNA fragment of GFP was amplified from pFA6aNat-Cut11-GFP (Aoki et al., 2011) using the primer pairs AGAAACAGTTTACACGTCTAATGGAGGAGGAAGTAAAGGAGAAGAACTTTTCA and AscI-TTATTTGTATAGTTCATCCATGCC (Pim1-fragment 4). The amplified DNA fragments 3 and 4 were mixed and used to amplify the combined fragment (Pim1-fragment 5) using the primers BamHI-GTTGAGTCTATTACCGGTGGTGAG and AscI-TTATTTGTATAGTTCATCCATGCC. The Pim1-fragment 5 was inserted into the BamHI-AscI site of pFA6aNat instead of the Pim1-fragment 1. To generate the mutated version of Pim1-3Flag or Pim1-GFP, DNA fragments 1 and 3 were amplified from the *pim1-R152C* genomic DNA.

To generate GFP-fused Lem2 (SJAG_01745.5), two DNA fragments were amplified from NIG2017 genomic DNA using the primer pairs BamHI-CTTACCCGTTTCCGAGTTCAATGA and AAAAGTTCTTCTCCTTTACTTCCTCCTCCATTCACCTGTGTTTCATTAA (Lem2-fragment 1), and PmeI-TTTTTTCCTTCTCCTGATGACTTT and EcoRI-CTATAGATTGCTTTTTCCCCTTTG (Lem2-fragment 2). A DNA fragment of GFP was amplified from pFA6aNat-Cut11-GFP (Aoki et al., 2011) using the primer pairs TTAATGAAACACAGGTGAATGGAGGAGGAAGTAAAGGAGAAGAACTTTT and AscI-TTATTTGTATAGTTCATCCATGCC (Lem2-fragment 3). The amplified DNA fragments 1 and 3 were mixed and used to amplify the combined fragment (Lem2-fragment 4) using the primers BamHI-CTTACCCGTTTCCGAGTTCAATGA and AscI-TTATTTGTATAGTTCATCCATGCC. Lem2-fragments 4 and 2 were inserted into the BamHI-AscI and PmeI-EcoRI sites, respectively, of the pFA6aNat-3Flag. The resulting plasmid, pLem2-GFP-Nat, was digested with BamHI and EcoRI, and then transformed into NIG2028 using electroporation.

To generate mCherry-fused Cut3 (SJAG_00871.5), two DNA fragments were amplified from NIG2017 genomic DNA using the primer pairs BglII-GAGGTTCTCAGACGTGATGAATTG and TCCTCCTCGCCCTTGCTCACTCCTCCTCCTATCGAGGCAGATTGCTTTT (Cut3-fragment 1), and PmeI-CACATAATCCTAATACCCGCATCC and EcoRI-CTGTTAATATCTTGGCATGCTAAG (Cut3-fragment 2). A DNA fragment of mCherry was amplified from pFA6aKan-H2A-mCherry (Aoki et al., 2013) using the primer pairs AAAAGCAATCTGCCTCGATAGGAGGAGGAGTGAGCAAGGGCGAGGAGGA and AscI-TTACTTGTACAGCTCGTCCAT (Cut3-fragment 3). The amplified DNA fragments 1 and 3 were mixed and used to amplify the combined fragment (Cut3-fragment 4) using the primers BglII-GAGGTTCTCAGACGTGATGAATTG and AscI-TTACTTGTACAGCTCGTCCAT. Cut3-fragments 4 and 2 were inserted into the BamHI-AscI and PmeI-EcoRI sites, respectively, of the pFA6aKan-3Flag. The resulting plasmid, pCut3-mCherry-Kan, was digested with BamHI and EcoRI, and then transformed into NIG2028 using electroporation.

To generate a glutamate mutation of threonine 19 in the CDK phosphorylation site located at 19-22 amino acids of Cut3, two DNA fragments were amplified from NIG2017 genomic DNA using the primer pairs SmaI-GTACTCAATTTCTCAACAAAGAAT and CGCGGCCTCTCAGCTCTGTCAGGTTCTGCATCTAGAATGGACGGTGTTG (Cut3-fragment 5), and CAACACCGTCCATTCTAGATGCAGAACCTGACAGAGCTGAGAGGCCGCG and AscI-TTATATCGAGGCAGATTGCTTTTC (Cut3-fragment 6). The amplified DNA fragments 5 and 6 were mixed and used to amplify the combined fragment (Cut3-fragment 7) using the primers SmaI-GTACTCAATTTCTCAACAAAGAAT and AscI-TTATATCGAGGCAGATTGCTTTTC. Cut3-fragment 7 was inserted into the SmaI-AscI site of the pFA6aKan-3Flag. To generate a glutamate mutation of threonine 45 in the CDK phosphorylation site located at 45-48 amino acids of Cut3, two DNA fragments were amplified from NIG2017 genomic DNA using the primer pairs SmaI-GTACTCAATTTCTCAACAAAGAAT and GCGTCCTTGTTGAATCGTACGGGTTCCTTCGTTGACGGTGACTCTGGAG (Cut3-fragment 8), and CTCCAGAGTCACCGTCAACGAAGGAACCCGTACGATTCAACAAGGACGC and AscI-TTATATCGAGGCAGATTGCTTTTC (Cut3-fragment 9). The amplified DNA fragments 8 and 9 were mixed and used to amplify the combined fragment (Cut3-fragment 10) using the primers SmaI-GTACTCAATTTCTCAACAAAGAAT and AscI-TTATATCGAGGCAGATTGCTTTTC. Cut3-fragment 10 was inserted into the SmaI-AscI site of the pFA6aKan-3Flag. Cut3-fragment 2 was inserted into the PmeI-EcoRI site of the pFA6aKan-3Flag. The resulting plasmids, pCut3-T19E-Kan and pCut3-T45E-Kan, were digested with SmaI and EcoRI, and then transformed into NIG2028 using electroporation. To generate double glutamate mutations of threonine 19 and 45, the DNA fragment 8 was amplified from pCut3-T19E-Kan.

To generate GFP-fused Rna1 (SJAG_04400.5), two DNA fragments were amplified from NIG2017 genomic DNA using the primer pairs BamHI-GTCGTCCGCATGGTGCAAAACGGT and AAAAGTTCTTCTCCTTTACTTCCTCCTCCAATAGATGCCTTAGCCATAG (Rna1-fragment 1), and PmeI-AGTAGCTTTTGCTGTCAGCAAAAC and EcoRV-GAAACAATTCAGGCTTGTCTGTGG (Rna1-fragment 2). A DNA fragment of GFP was amplified from pFA6aNat-Cut11-GFP (Aoki et al., 2011) using the primer pairs CTATGGCTAAGGCATCTATTGGAGGAGGAAGTAAAGGAGAAGAACTTTT and AscI-TTATTTGTATAGTTCATCCATGCC (Rna1-fragment 3). The amplified DNA fragments 1 and 3 were mixed and used to amplify the combined fragment (Rna1-fragment 4) using the primers BamHI-GTCGTCCGCATGGTGCAAAACGGT and AscI-TTATTTGTATAGTTCATCCATGCC. Lem2-fragments 4 and 2 were inserted into the BamHI-AscI and PmeI-EcoRV sites, respectively, of the pFA6aNat-3Flag. The resulting plasmid, pRna1-GFP-Nat, was digested with BamHI and EcoRV, and then transformed into NIG2028 using electroporation.

To generate GFP-fused Nup85 (SJAG_00471.5), two DNA fragments were amplified from NIG2017 genomic DNA using the primer pairs BamHI-GGCTTGTTTACATATGAATAAGGC and AAAAGTTCTTCTCCTTTACTTCCTCCTCCCTTTAACAAGAACAATCTTG (Nup85-fragment 1), and PmeI-GATACGTTTCACTGTCCCATATAA and EcoRI-CTTTTACAAAAACTCGATGAATCC (Nup85-fragment 2). A DNA fragment of GFP was amplified from pFA6aNat-Cut11-GFP (Aoki et al., 2011) using the primer pairs CAAGATTGTTCTTGTTAAAGGGAGGAGGAAGTAAAGGAGAAGAACTTTT and AscI-TTATTTGTATAGTTCATCCATGCC (Nup85-fragment 3). The amplified DNA fragments 1 and 3 were mixed and used to amplify the combined fragment (Rna1-fragment 4) using the primers BamHI-GGCTTGTTTACATATGAATAAGGC and AscI-TTATTTGTATAGTTCATCCATGCC. Nup85-fragments 4 and 2 were inserted into the BamHI-AscI and PmeI-EcoRI sites, respectively, of the pFA6aNat-3Flag. The resulting plasmid, pNup85-GFP-Nat, was digested with BamHI and EcoRI, and then transformed into NIG2028 using electroporation.

To generate a deletion mutant of *nup61^+^* (SJAG_04284.5), two DNA fragments were amplified from NIG2017 genomic DNA using the primer pairs BamHI-CAGAAATGGGTATTTGAAGAAGAG and AscI-TTTGTTCCTTAGGACTAAATTTTG (Nup61-fragment 1), and PmeI-GATTTACATCTAACACACATACAA and EcoRI-CAGACACGAAATTCGTTGAAGAGT (Nup61-fragment 2). Nup85-fragments 1 and 2 were inserted into the BamHI-AscI and PmeI-EcoRI sites, respectively, of the pFA6aNat-3Flag. The resulting plasmid, pΔnup61-Kan, was digested with BamHI and EcoRI, and then transformed into NIG2028 using electroporation.

To generate the fusion protein of Pim1-Ely5-GFP, three DNA fragments were amplified from NIG2017 genomic DNA using the primer pairs BamHI-CGAACAACGAGCGAAGACGAAACG and TGGAACTGCTCAGTGTTCATTCCTCCTCCATTAGACGTGTAAACTGTTT (Pim1-Ely5-fragment 1), and AAACAGTTTACACGTCTAATGGAGGAGGAATGAACACTGAGCAGTTCCA and AAAAGTTCTTCTCCTTTACTTCCTCCTCCAGGAACCATCGTTTTAATAG (Pim1-Ely5-fragment 2), and AscI-ATATTACAAATAAATCAAGTAACC and BglII-GCGGCCGCATTCGGGTTCTGCGTCTCCGAAAG (Pim1-Ely5-fragment 3). A DNA fragment of GFP was amplified from pFA6aNat-Cut11-GFP (Aoki et al., 2011) using the primer pairs CTATTAAAACGATGGTTCCTGGAGGAGGAAGTAAAGGAGAAGAACTTTT and AscI-TTATTTGTATAGTTCATCCATGCC (Pim1-Ely5-fragment 4). The amplified DNA fragments 1, 2 and 3 were mixed and used to amplify the combined fragment (Pim1-Ely5-fragment 5) using the primers BamHI-CGAACAACGAGCGAAGACGAAACG and AscI-TTATTTGTATAGTTCATCCATGCC. Nup85-fragments 5 was digested with BamHI and AscI, and Nup85-fragments 2 was digested with AscI and BglII. These two digested DNA fragments were cloned into the BamHI site of pSJU11 plasmid (Aoki et al., 2010). The resulting plasmid, pSJU11-Pim1-Ely5, was transformed into NIG8002 using electroporation.

The method used to construct GFP-AHDL was modified from those used in previous reports (Pidoux and Armstrong, 1992; Yam et al., 2011). The artificial gene of GFP-AHDL was replaced by *ade6^+^* in the genome of *S. japonicus*. To generate GFP-AHDL, two DNA fragments were amplified from the *ade6^+^* locus of NIG2017 genomic DNA using the primer pairs BamHI-CGTGAAGCTCAAAGCGATTGCAAA and ATTGGTTGAATTGTAAAAGTCATGTTTAGTAATTCAAAAAACAATAAG (AHDL-fragment 1), and PmeI-ATACAACAAGCTAGCTTAAGTGAA and EcoRI-CCTTGGAGAGAACGTATCTGGACT (AHDL-fragment 2). In addition, a DNA fragment from the 5′ untranslated region and N-terminal 75 bp of *bip1^+^* was amplified from 972 genomic DNA in *S. pombe* using the primer pair CTTATTGTTTTTTGAATTACTAAACATGACTTTTACAATTCAACCAAT and AGTGAAAAGTTCTTCTCCTTTACTACTAGCAAAAGCCATAGGTAGGAG (AHDL-fragment 3). Moreover, a DNA fragment of GFP was amplified from pFA6aKan-Cut11-GFP (Aoki et al., 2011) using the primer pair CTCCTACCTATGGCTTTTGCTAGTAGTAAAGGAGAAGAACTTTTCACT and AscI-TTACAGGTCGTGTGCTTTGTATAGTTCATCCATGC (AHDL-fragment 4). The amplified DNA fragments 1, 3 and 4 were mixed and used to amplify the combined fragment (AHDL-fragment 5) using the primers BamHI-CGTGAAGCTCAAAGCGATTGCAAA and AscI-TTACAGGTCGTGTGCTTTGTATAGTTCATCCATGC. AHDL-fragments 5 and 2 were inserted into the BamHI-AscI and PmeI-EcoRI sites, respectively, of the pFA6aNat-3Flag. The resulting plasmid, pGFP-AHDL-Nat, was digested with BamHI and EcoRI, and then transformed into NIG2028 using electroporation.

Genetic information of *cut14^+^* (SJAG_03832) was annotated in the NCBI server. To generate mCherry-fused Cut14 (SJAG_03832.5), two DNA fragments were amplified from NIG2017 genomic DNA using the primer pairs BamHI-GCTTAGATCAATTCAAACGAAGTG and TCCTCCTCGCCCTTGCTCACTCCTCCTCCTTTTGCTTGTACAACTGAAG (Cut14-fragment 1), and PmeI-TACCCTACTACATGACTTGAGTCA and EcoRI-CTTCACAATTCCGTAAAATGTCAC (Cut14-fragment 2). A DNA fragment of mCherry was amplified from pFA6aKan-H2A-mCherry (Aoki et al., 2013) using the primer pair CTTCAGTTGTACAAGCAAAAGGAGGAGGAGTGAGCAAGGGCGAGGAGGA and AscI-TTACTTGTACAGCTCGTCCAT (Cut14-fragment 3). The amplified DNA fragments 1 and 3 were mixed and used to amplify the combined fragment (Cut14-fragment 4) using the primers BamHI-GCTTAGATCAATTCAAACGAAGTG and AscI-TTACTTGTACAGCTCGTCCAT. Cut3-fragments 4 and 2 were inserted into the BamHI-AscI and PmeI-EcoRI sites, respectively, of the pFA6aKan-3Flag. The resulting plasmid, pCut14-mCherry-Kan, was digested with BamHI and EcoRI, and then transformed into NIG2028 using electroporation.

## Supplementary Material

Supplementary information
