# Supplementary material for: Release of condensin from mitotic chromosomes requires the Ran-GTP gradient in the reorganized nucleus
Source: Biol Open. 2017 Sep 27;6(11):1614–28. doi: 10.1242/bio.027193 (PMC5703609; doi:10.1242/bio.027193)
Supplement: Supplementary information [file biolopen-6-027193-s1.pdf]

Supplementary table 1. Strains used in the present study

Name	Genotype	Reference
NIG2028	*h^- mat-P2028*	Furuya and Niki, 2009
NIG8001	*h^- mat-P2028 pim1-R152C*	This study
NIG8002	*h^- mat-P2028 pim1-R152C ura4-D3*	This study
NIG8003	*h^- mat-P2028 hta1-mCherry:kan*	This study
NIG8004	*h^- mat-P2028 hta1-mCherry:kan pim1-R152C*	This study
NIG8896	*h^- mat-P2028 cut11-GFP:nat hta1-mCherry:kan*	Aoki et al., 2013
NIG8005	*h^- mat-P2028 cut11-GFP:nat hta1-mCherry:kan pim1-R152C*	This study
NIG8006	*h^+ mat-P2017 lem2-GFP:nat hta1-mCherry:kan*	This study
NIG8007	*h^- mat-P2028 lem2-GFP:nat hta1-mCherry:kan pim1-R152C*	This study
NIG8008	*h^- mat-P2028 rna1-GFP:nat hta1-mCherry:kan*	This study
NIG8009	*h^- mat-P2028 rna1-GFP:nat hta1-mCherry:kan pim1-R152C*	This study
NIG8010	*h^- mat-P2028 nup85-GFP:nat*	This study
NIG8011	*h^- mat-P2028 nup85-GFP:nat pim1-3Flag:nat*	This study
NIG8012	*h^- mat-P2028 nup85-GFP:nat $pim1^{R152C}$-3Flag:nat*	This study
NIG8013	*h^- mat-P2028 pim1-GFP:nat hta1-mCherry:kan*	This study
NIG8014	*h^- mat-P2028 pim1-GFP:nat hta1-mCherry:kan nup61::kan*	This study
NIG8015	*h^- mat-P2028 $pim1^{R152C}$-GFP:nat hta1-mCherry:kan*	This study
NIG8016	*h^+ mat-P2017 hta1-mCherry:nat cut3-GFP:kan*	This study
NIG8018	*h^- mat-P2028 hht3-GFP:kan cut3-mCherry:kan*	This study

Biology Open • Supplementary information

NIG8019	*h^+ mat-P2017 hht3-GFP:kan cut3-mCherry:kan nup61::kan*	This study
NIG8020	*h^- mat-P2028 hht3-GFP:kan cut3-mCherry:kan pim1-R152C*	This study
NIG8021	*h^+ mat-P2017 cut11-GFP:nat cut3-mCherry:kan*	This study
NIG8022	*h^- mat-P2028 cut11-GFP:nat cut3-mCherry:kan pim1-R152C*	This study
NIG8023	*h^- mat-P2028 cut3-T19E:kan*	This study
NIG8024	*h^- mat-P2028 cut3-T45E:kan*	This study
NIG8025	*h^- mat-P2028 cut3-T19ET45E:kan*	This study
NIG8026	*h^+ mat-P2017 cut3-T19E:kan pim1-R152C*	This study
NIG8027	*h^+ mat-P2017 cut3-T45E:kan pim1-R152C*	This study
NIG8028	*h^+ mat-P2017 cut3-T19ET45E:kan pim1-R152C*	This study
NIG8029	*h^- mat-P2028 hta1-mCherry:nat cut3-T19E:kan*	This study
NIG8030	*h^+ mat-P2017 hta1-mCherry:nat cut3-T19E:kan pim1-R152C*	This study
NIG8031	*h^- mat-P2028 pim1-GFP:nat*	This study
NIG8036	*h^- mat-P2028 hta1-mCherry:nat cut3-GFP:kan pim1-R152C*	This study
NIG8037	*h^+ mat-P2017 hht3-GFP:kan cut3-T19E-mCherry:kan*	This study
NIG8039	*h^- mat-P2028 hta1-mCherry:nat pim1-R152C ura4-D3*	This study

Biology Open • Supplementary information

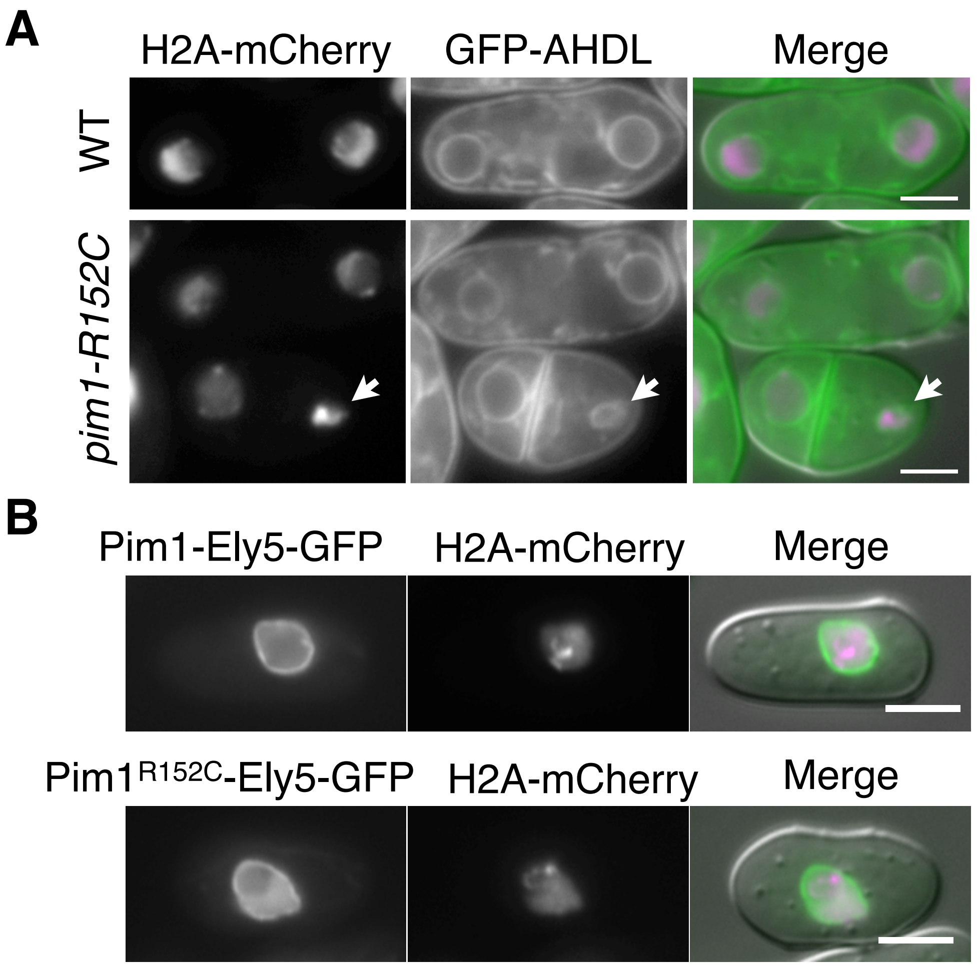

Supplementary figure 1. Observation of GFP-AHDL, Pim1-Ely5-GFP, and Pim1^{R152C}-Ely5-GFP. (A) Simultaneous observation of H2A-mCherry and GFP-AHDL was performed. Strains of *h*$^{+}$ *mat-P2017 GFP-AHDL:nat hta1-mCherry:kan* (NIG8035) and *h*$^{-}$ *mat-P2028 GFP-AHDL:nat hta1-mCherry:kan pim1-R152C* (NIG8038) were cultivated to log phase at 30˚C and shifted to 37˚C for 2 h. GFP-AHDL and H2A-mCherry are observed in the WT (n=483) or *pim1-R152C* (n=729). Signals of GFP-AHDL were observed in 71% around the hyper-condensed chromatin (n=65). Signals of GFP-AHDL and H2A-mCherry are shown in green and magenta, respectively. Arrows indicate the hyper-condensed chromatin. Bar = 5μm. (B) Localizations of Pim1-Ely5-GFP and Pim1^{R152C}-Ely5-GFP were observed. Transformants of pSJU11-pim1-ely5-GFP (n=154) and pSJU11-pim1^{R152C}-ely5-GFP (n=166) in Fig. 2D were cultivated to log phase in YE (+Ade, Ura) medium at 30°C and observed using a phase contrast microscope. Two independent experiments were performed in each experiment. Bar = 5 μm.

Supplementary fig. 1

Biology Open • Supplementary information

Biology Open (2017): doi:10.1242/bio.027193: Supplementary information

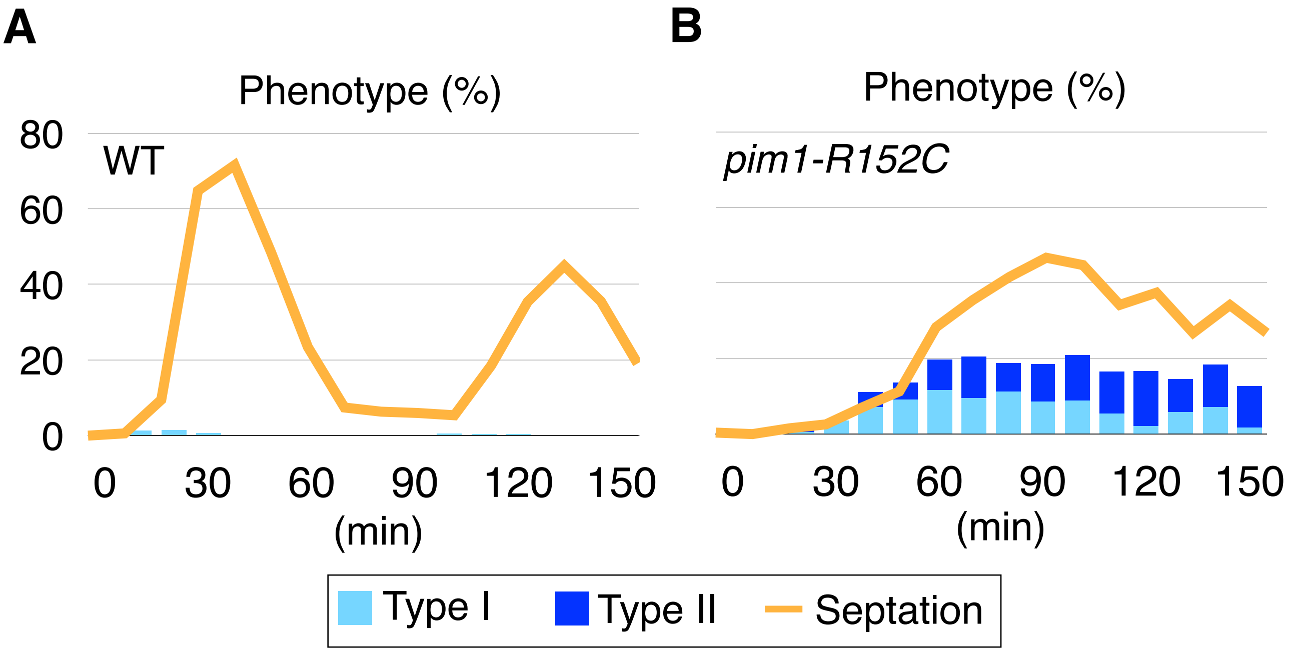

Supplementary figure 2. Synchronization experiments show that decrease of Pim1^{R152C}-GFP was followed by hyper-condensation of chromatin. Synchronization experiments were performed once in the WT (A) or *pim1-R152C* (B), as shown in Materials and Methods. Strains of *h^- mat-P2028 pim1-GFP:nat cut11-mCherry:nat* (NIG8040) and *h^- mat-P2028 pim1^{R152C}-GFP:nat cut11-mCherry:nat* (NIG8041) were used for this purpose. In concert with Fig. 3C and 3D, type I cells are defined as binucleated cells having a normal sized nuclear envelope with decreased Pim1^{R152C}-GFP signal. Type II cells are defined as binucleated cells having a compacted sized nuclear envelope without Pim1^{R152C}-GFP signal. The combined percentage of type I and type II cells reached about 21% of total cells at 100 min in pim1-R152C. Sky blue bar: the frequency of the type I cell; blue bar: the frequency of the type II cell; orange line: the frequency of septated cell. Number of cells analyzed at each time point is following. In the WT, 0min (n=276), 10min (n=167), 20min (n=305), 30min (n=205), 40min (n=275), 50min (n=297), 60min (n=213), 70min (n=188), 80min (n=315), 90min (n=200), 100min (n=241), 110min (n=297), 120min (n=299), 130min (n=280), 140min (n=194), 150min (n=288). In pim1-R152C, 0min (n=278), 10min (n=309), 20min (n=426), 30min (n=338), 40min (n=322), 50min (n=297), 60min (n=179), 70min (n=219), 80min (n=314), 90min (n=278), 100min (n=325), 110min (n=389), 120min (n=300), 130min (n=271), 140min (n=368), 150min (n=329).

Supplementary fig. 2

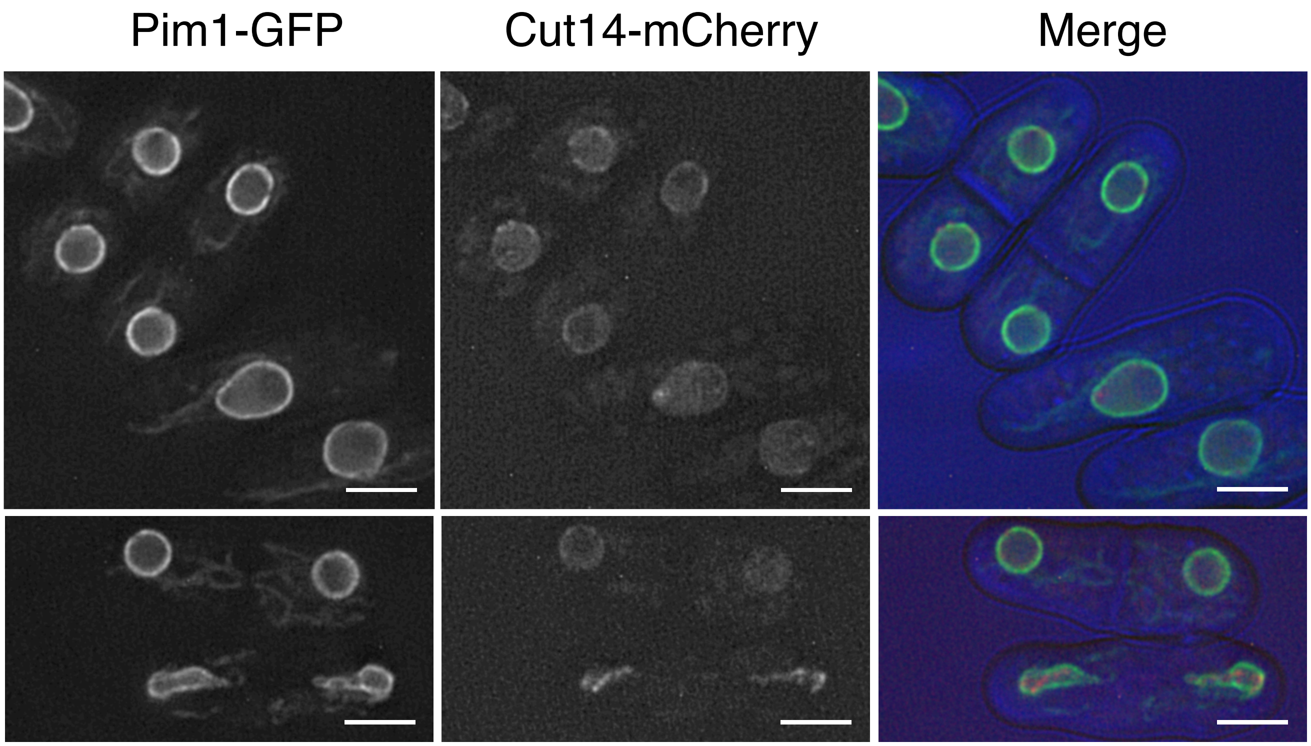

Supplementary figure 3. Simultaneous observation of Pim1-GFP and Cut14-mCherry. A strain of *h⁻ mat-P2028 pim1-GFP:nat cut14-mCherry:kan* (NIG8017) was cultivated to log phase in YE (+Ade, Ura) medium at 30°C and observed using a DeltaVision microscope (n=161). Green, red, and blue indicate Pim1-GFP, Cut14-mCherry, and DIC signals, respectively. Cut14-mCherry was localized around the nuclear envelope in binucleated cells. Two independent experiments were performed. Bar = 5 μm.

Supplementary fig. 3

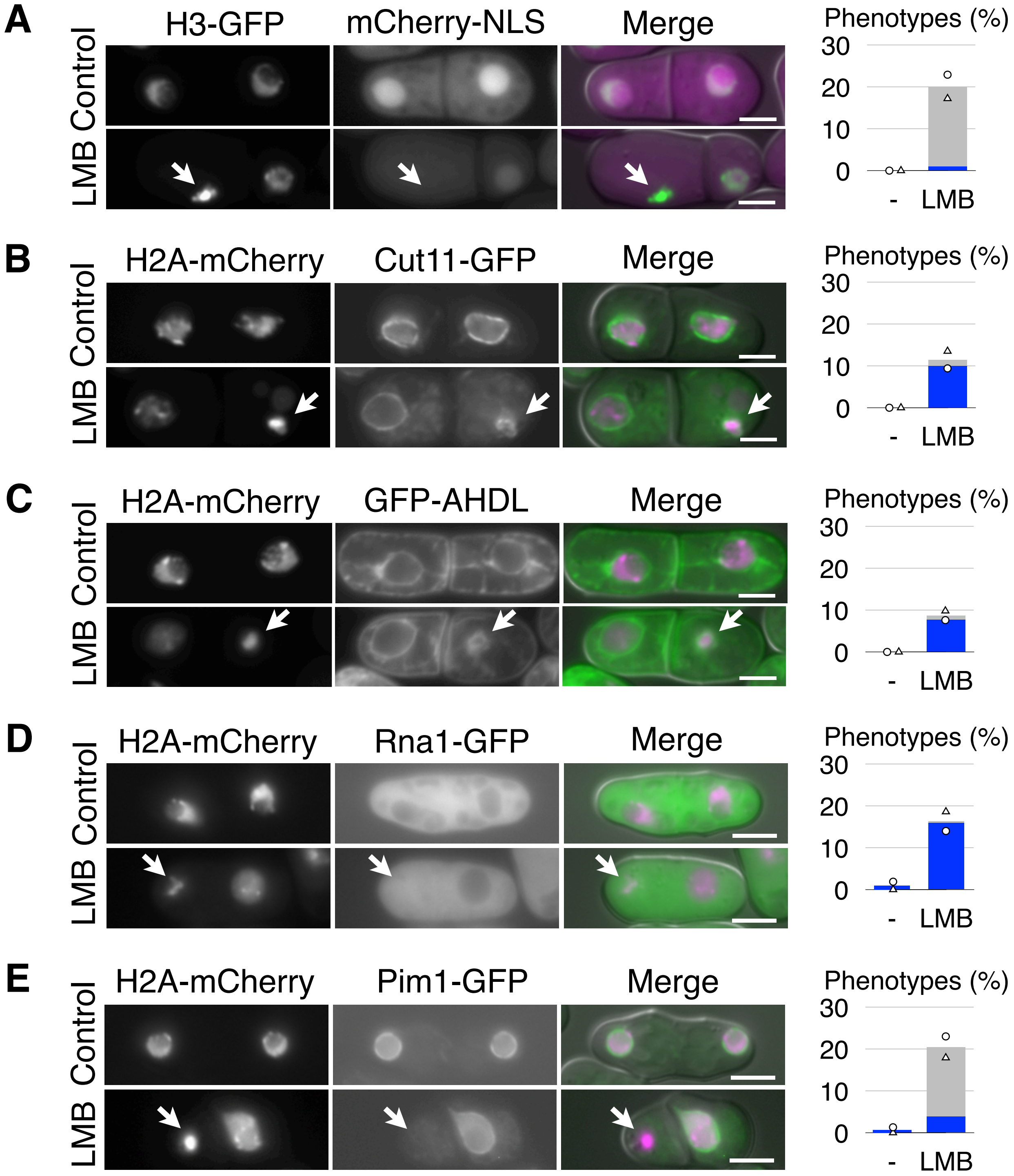

Supplementary fig. 4

Supplementary figure 4. Localization of mCherry-NLS, Cut11-GFP, GFP-AHDL, Rna1-GFP, and Pim1-GFP in leptomycin B (LMB)-treated cells. Strains of *h^- mat-P2028 hht3-GFP:kan ura4-D3 /pSJU11-mCherry-NLS* (NIG8032), *h^- mat-P2028 cut11-GFP:nat hta1-mCherry:kan* (NIG8896), *h^+ mat-P2017 GFP-AHDL:nat hta1-mCherry:kan* (NIG8035), *h^- mat-P2028 rna1-GFP:nat hta1-mCherry:kan* (NIG8008), and *h^- pim1-GFP:nat hta1-mCherry:kan* (NIG8013) were cultivated to log phase in YE (+Ade, Ura) medium at 30°C and treated with 4μM LMB or ethanol (control). After the treatment, the cells were cultivated at 30°C for 4 h and observed using a phase contrast microscope. (A) Following LMB treatment, hyper-condensed chromatin was observed in 20.7% of cells (n=693 cells). In addition, the mCherry-NLS signal had disappeared from 95% of the hyper-condensed nuclei (grey). (B) Following LMB treatment, hyper-condensed chromatin was observed in 12.8% of cells (n=598 cells). The Cut11-GFP signal was observed in 86% of the hyper-condensed nuclei (blue). (C) Following LMB treatment, hyper-condensed chromatin was observed in 8.7% of cells (n=649 cells). In addition, the GFP-AHDL signal was observed in all of the hyper-condensed nuclei (blue). (D) Following LMB treatment, hyper-condensed chromatin was apparent in 16.3% of cells (n=216 cells). The Rna1-GFP signal was observed in 98% of the hyper-condensed nuclei (blue). (E) Following LMB treatment, hyper-condensed chromatin was observed in 20.5% of cells (n=234 cells). In addition, the Pim1-GFP signal had disappeared in 81.2% of the hyper-condensed nuclei (grey). The pSJU11-mCherry-NLS construct was previously reported in Aoki et al., 2011. In A, B, C, D, E, each symbol shows results from two independent experiments, and the bar graphs show the mean of these experiments. Bar = 5 μm.
